# MALDI-TOF-MS reveals differential *N*-linked plasma- and IgG-glycosylation profiles between mothers and their newborns

**DOI:** 10.1038/srep34001

**Published:** 2016-09-26

**Authors:** Bas C. Jansen, Albert Bondt, Karli R. Reiding, Sicco A. Scherjon, Gestur Vidarsson, Manfred Wuhrer

**Affiliations:** 1 Center for Proteomics and Metabolomics, Leiden University Medical Center, 2300 RC Leiden, The Netherlands; 2 Department of Rheumatology, Leiden University Medical Center, 2300 RC Leiden, The Netherlands; 3 Department of Obstetrics and Gynaecology, University Medical Center Groningen, 9713 GZ Groningen, The Netherlands; 4 Department of Experimental Immunohematology, Sanquin Research, Amsterdam, The Netherlands

## Abstract

During pregnancy, the mother provides multiple nutrients and substances to the foetus, with maternal immunoglobulin G (IgG) being actively transported to the foetus. Newborns depend on maternal IgG for immune-protection in their first months. The glycosylation of IgG has been shown to influence its dynamics, *e.g.* receptor binding. While minor differences in IgG glycosylation have been found between IgG derived from maternal blood and umbilical cord blood (UC) of newborn children, the differential glycosylation of maternal and UC plasma has hitherto not been studied. Here, we studied the *N*-glycosylation of IgG and total plasma proteome of both maternal and UC plasma of 42 pairs of mothers and newborn children. A total of 37 *N*-glycans were quantified for IgG and 45 for the total plasma *N*-glycome (TPNG). The study showed slightly higher levels of galactosylation for UC IgG than maternal IgG, confirming previous results, as well as lower bisection and sialylation. Furthermore, the TPNG results showed lower values for galactosylation and sialylation, and higher values for fucosylation in the UC plasma. In conclusion, this study presents some novel insights into IgG glycosylation differences as well as the first broad overview of the differential plasma glycosylation between mothers and newborns.

Pregnancy induces major changes in many physiological and immunological processes^1^,^2^. For example, the concentration of immunoglobulin G (IgG) – the most abundant glycoprotein in humans with an average concentration of 10.6 mg/mL – decreases during pregnancy from 9.35 mg/mL (7–17 weeks) to 7.80 mg/mL (34–38 weeks)^3^. This decrease is believed to be caused by the active transfer of IgG to the developing foetus in order to establish a functional humoral immune system, as the foetus itself is only capable of producing minute amounts of IgG. Another contributing factor is dilution of plasma protein concentrations due to a 40–50% increase in plasma volume^4^,^5^,^6^. IgG represent the only class of glycoproteins known to be transported across the placenta.

IgG is an important player in the human humoral immune system, in which it is involved in antigen binding, antibody dependent cellular cytotoxicity, and the activation of the complement system^7^. The dynamics of IgG are heavily influenced by its glycosylation, *e.g.* IgG with digalactosylated glycans on the Fc portion binds stronger to Fc-gamma-receptor (FcγR) IIa, while afucosylated IgG1 binds FcγRIIIa and FcγRIIIb with up to 50 fold increased affinity^8^,^9^. The neonatal Fc receptor (FcRn) is an IgG receptor that has been found on the placenta and has been linked to the transfer of IgG from the maternal to the foetal circulation^10^. The FcRn binds to the outward-facing part of the C_H_2-C_H_3 region of IgG, with the inner facing part being occupied by the conserved *N*-glycosylation site^11^. Because of this, it has been suggested that the FcRn receptor might prefer certain IgG glycoforms resulting in preferred transport of certain IgG glycoforms^12^,^13^,^14^. In support of this, a recent study showed that deglycosylated IgG has a slight diminished binding to FcRn, with digalactosylated IgG showing better binding than monogalactosylated and agalactosylated species^15^, other research including *in vivo* pharmacological studies show an impact of the glycan on the half-life mediated by FcRn^16^,^17^,^18^. Furthermore, agalactosylated IgG has also been reported to be transported equally well across the placenta^19^, suggesting that glycosylation of IgG does not strongly affect FcRn mediated biology.

Notably, hardly anything is known about the glycosylation of other plasma proteins of the foetus. Studying the total plasma *N*-glycome (TPNG), referring to the overall glycosylation profile as obtained by releasing *N*-glycans from all proteins present in plasma, could provide novel insights on top of a single protein approach. The validity of this approach in the field of glycomics has been proven by several recent publications^20^,^21^.

Here, we present a comparison between maternal and foetal IgG and total plasma glycosylation using plasma from the circulation of 42 mothers and the umbilical cord of their newborn children (UC)^14^. We performed the comparison using a recently developed derivatisation technique to distinguish between α2,6- and α2,3-linked sialic acids, and a high-throughput data processing package^22^,^23^.

## Results

**Figure 1:**
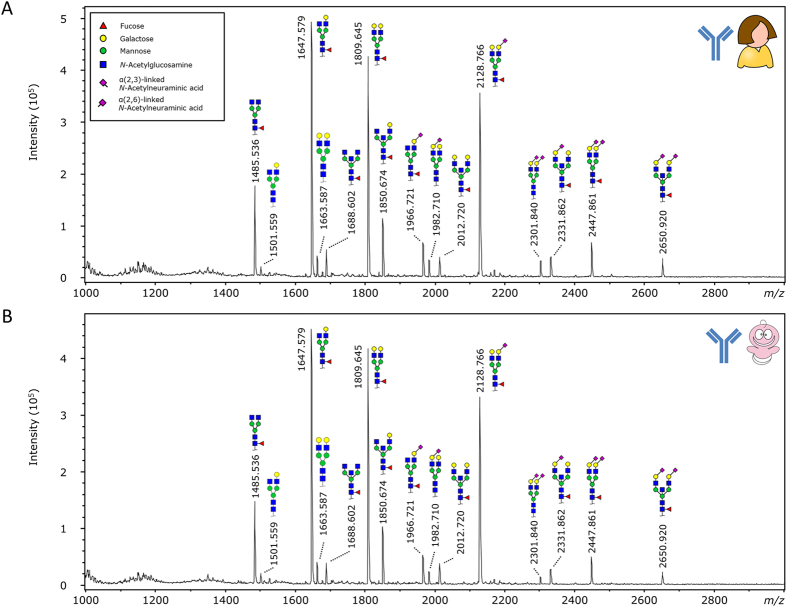
IgG glycan MALDI-TOF-MS spectra of maternal and UC origin. (**A**) Maternal IgG glycome and (**B**) UC IgG glycome. IgG was captured from plasma, followed by a glycan release and derivatisation as described in literature^43^. The glycan structures that are shown are based on the most common glycan structures in healthy human IgG^47^. The displayed maternal and UC samples are of a matching pair.

Released glycans from purified IgG and total plasma from paired maternal and UC origin were measured by matrix-assisted laser desorption/ionisation (MALDI)-time-of-flight (TOF)-mass spectrometry (MS) (Fig. 1).

### Technical and Biological Variation

A total of 6 measurements of commercially available pooled plasma was used to determine the technical variation. Of these, the coefficient of variation (CV) for the main glycan was determined after normalisation to the total analyte area. Glycan compositions are displayed using a single letter notation, *i.e.* hexose (H), *N-*acetylhexosamine (N), fucose (F), ethyl esterified *N*-acetylneuraminic acid (E) and lactonised *N*-acetylneuraminic acid (L). The main TPNG composition H5N4E2 showed a CV of 2.8%, while IgG-specific H4N4F1 showed a CV of 8.5%. Furthermore, the biological variation was determined by calculating the CV for the main glycan measured in both the maternal and UC TPNG and IgG samples. The observed CVs in the cohort reflect a combination of both biological and technical variation, being 16.2% for maternal plasma, 11.2% for UC plasma, 12.1% for maternal IgG and 13.6% for UC IgG.

Lastly, the maximum potential contamination of the IgG glycosylation by non-IgG contaminants was estimated on the basis of the relative abundance of the glycan species corresponding to the main glycan in TPNG^24^. This glycan H5N4E2 showed on average a relative abundance (1%) indicating that a potential aspecific contamination with other plasma proteins is minor if noticeable at all.

### IgG Glycosylation Comparison

**Figure 2:**
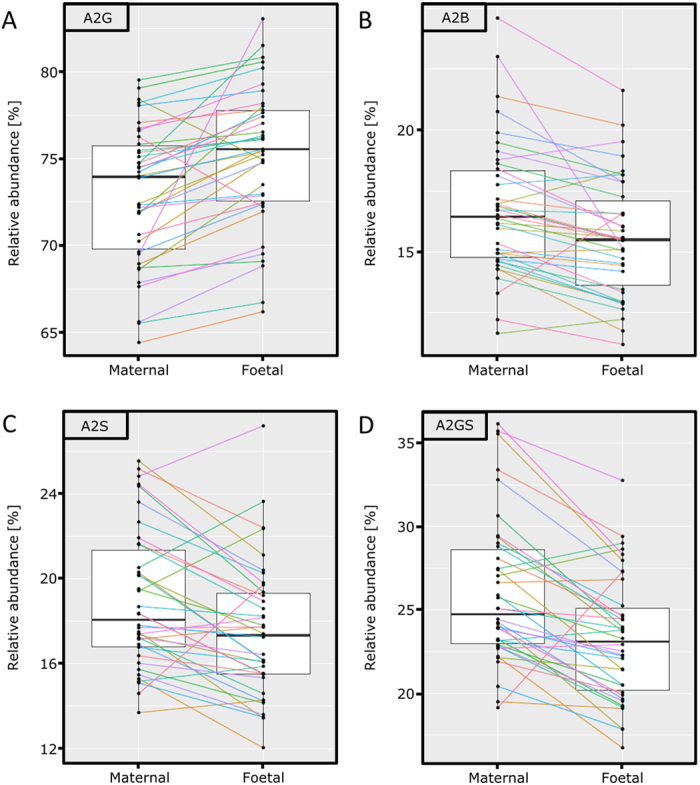
Glycosylation differences between maternal and UC IgG. The level in both maternal and UC IgG of each individual pair and boxplots for the entire group are displayed. The figure displays 4 calculated derived traits; (**A**) % galactosylation of diantennary compositions, (**B**) % bisection of diantennary compositions, (**C**) % sialylation of diantennary compositions and (**D**) % sialylation per galactose of diantennary compositions.

A total of 37 glycoforms was quantified from the purified IgG (Fig. 1; Supplementary Table S1), followed by a Wilcoxon signed-rank test for comparing maternal and UC IgG glycosylation features. Multiple testing correction was performed by Bonferroni adjustment of the significance threshold. This showed for the UC lower levels of the agalactosylated and some monogalactosylated glycans H3N4F1 (mother versus UC: 6.3% *vs.* 4.8%; *p* = 1.4∙10^−8^), H3N5F1 (2.3% *vs.* 1.9%; *p* = 1.2∙10^−5^), H4N4F1E1 (2.4% *vs.* 1.9%; *p* = 4.9∙10^−7^), while other monogalactosylated and digalactosylated structures showed higher levels in UC: H4N5F1 (mother versus UC: 5.6% *vs.* 6.1%, *p* = 1.2∙10^−5^), H5N4 (2.0% *vs.* 2.4%, *p* = 5.0∙10^−5^) and H5N4F1 (23.2% *vs.* 25.8%, *p* = 2.6∙10^−6^). A total of 7 derived traits reflecting specific glycosylation features were calculated from the individual glycans (Supplementary Table S2). These derived traits also showed a higher level of galactosylation for UC (A2G; mother versus UC: 73.0% *vs.* 75.2%; *p* = 8.6∙10^−8^; Fig. 2A), and conversely a lower level of agalactosylation (TaGal, 9.8% *vs.* 7.8%; *p* = 3.6∙10^−6^), matching the previously described change in agalactosylation^12^,^13^. Three additional traits were lower in UC, which were bisection (A2B; 16.7% *vs.* 15.6%; *p* = 3.9∙10^−5^; Fig. 2B), sialylation (A2S; 19.0% *vs.* 17.5%; *p* = 2.1∙10^−3^; Fig. 2C) and sialylation per galactose (A2GS; 26.1% *vs.* 23.3%; p-value = 5.0∙10^−5^; Fig. 2D). All mentioned derived traits were calculated following normalisation to the total area of diantennary complex type glycan compositions. A summary of the derived glycosylation trait results is presented in Table 1. The box plots presented for the derived traits (Fig. 2) also show the differences between maternal and UC plasma on a pair-wise basis, as indicated with the coloured lines.

### TPNG Comparison

**Figure 3:**
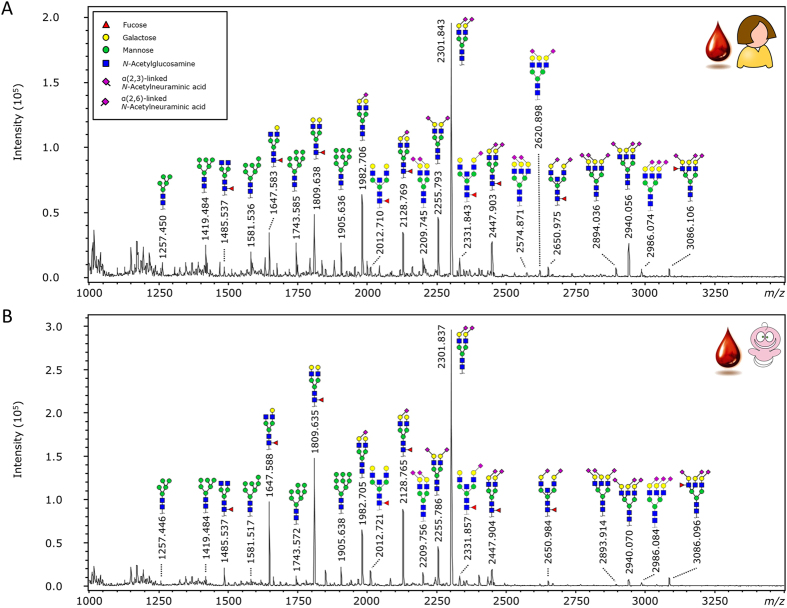
TPNG MALDI-TOF-MS spectra of maternal and UC origin. (**A**) Maternal TPNG and (**B**) UC TPNG. The glycans were released, derivatised and measured as described in literature^22^. The glycan structures that are shown are based on the most common glycan structures in healthy human plasma^22^,^24^,^48^. The displayed maternal and UC samples are of a matching pair.

A total of 45 glycoforms was quantified from the TPNG of maternal and UC origin, to investigate the potential differences between the glycomes (Fig. 3). All individual glycan results are presented in Supplementary Table S3, these were used to calculate 71 derived traits as described in Supplementary Table S4.

### General Traits

**Figure 4:**
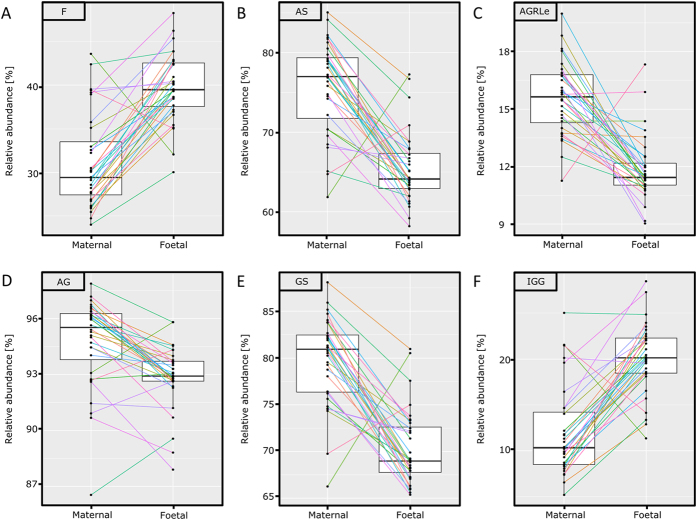
Glycosylation differences between maternal and UC TPNG. The differences in TPNG glycosylation are presented for each individual pair as well as the entire group. Six relevant derived traits are presented, namely (**A**) % fucosylation, (**B**) % sialylation per antenna, (**C**) ratio of α2,3-linked/α2,6-linked sialylation per galactose of complex compositions, (**D**) % galactosylation per antenna, (**E**) % sialylation per galactose and (**F**) % glycan compositions unique to IgG, being the non sialylated fucosylated diantennary glycans.

UC plasma showed higher levels of hybrid glycans (Hy; mother versus UC: 0.6% *vs.* 0.9%; *p* = 5.4∙10^−4^), diantennary glycans (A2; 77.1% *vs.* 85.1%; *p* = 1.0∙10^−5^), fucosylation (F; 31.1% *vs.* 39.7%; *p* = 6.0∙10^−6^; Fig. 4A) and glycan compositions supposedly unique to IgG (IGG; 11.9% *vs.* 20.1%; *p* = 1.1∙10^−5^; Fig. 4F). Glycan trait levels of UC were lower for galactosylation per antenna (AG; 94.8% *vs.* 92.9%; *p* = 1.1∙10^−4^; Fig. 4D), sialylation per antenna (AS; 75.8% *vs.* 65.5%; *p* = 1.3∙10^−5^; Fig. 4B), α2,3-linked sialylation per antenna (AL; 12.0% *vs.* 7.9%; *p* = 9.4∙10^−6^), α2,6-linked sialylation per antenna (AE; 63.8% *vs.* 57.6%; *p* = 5.0∙10^−5^), sialylation per galactose (GS; 79.7% *vs.* 70.4%; *p* = 1.3∙10^−5^; Fig. 4E), α2,3-linked sialylation per galactose (GL; 12.4% *vs.* 8.4%; *p* = 9.4∙10^−6^), α2,6-linked sialylation per galactose (GE; 67.3% *vs.* 62.0%; *p* = 6.3∙10^−5^) and the percentage of sialylation on complex compositions being α2,3-linked (AGRle; 15.6% *vs.* 11.8%; *p* = 1.1∙10^−5^; Fig. 4C). A summary of all derived traits is presented in Table 2.

### Diantennary Traits

For fucosylation (A2F; 34.2% *vs.* 41.4%; *p* = 2.8∙10^−5^) and the percentage of sialylation on fucosylated compositions being α2,3-linked (A2FGRle; 18.4% *vs.* 21.9%; *p* = 5.1∙10^−4^) higher levels in the UC plasma were observed. In contrast, lower levels were observed for bisection of fucosylated compositions (A2FB; 21.0% *vs.* 15.8%; *p* = 7.4∙10^−5^), sialylation (A2S; 72.6% *vs.* 63.6%; 2.8∙10^−5^), sialylation of fucosylated compositions (A2FS; 41.5% *vs.* 29.8%; *p* = 1.2∙10^−5^), α2,3-linked sialylation (A2L; 7.9% *vs.* 6.0%; *p* = 9.4∙10^−6^), α2,3-linked sialylation of non-fucosylated compositions (A2F0L; 8.2% *vs.* 5.7%; *p* = 2.8∙10^−5^), α2,6-linked sialylation (A2E; 64.7% *vs.* 57.6%; *p* = 6.3∙10^−5^), α2,6-linked sialylation of fucosylated compositions (A2FE; 33.8% *vs.* 23.2%; *p* = 3.0∙10^−5^), sialylation per galactose (A2GS; 77.3% *vs.* 68.9%; *p* = 2.0∙10^−5^), sialylation per galactose of fucosylated compositions (A2FGS; 47.7% *vs.* 35.0%; *p* = 1.0∙10^−5^), α2,3-linked sialylation per galactose (A2GL; 8.4% *vs.* 6.5%; *p* = 8.6∙10^−6^), α2,3-linked sialylation per galactose of non-fucosylated compositions (A2F0GL; 8.4% *vs.* 5.9%; *p* = 2.6∙10^−5^), α2,6-linked sialylation per galactose (A2GE; 68.9% *vs.* 62.4%; *p* = 3.9∙10^−5^), α2,6-linked sialylation per galactose of fucosylated compositions (A2FGE; 38.9% *vs.* 27.3%; *p* = 3.3∙10^−5^), the percentage of sialic acids on diantennary compositions being α2,3-linked (A2GRle; 10.9% *vs.* 9.5%; *p* = 1.1∙10^−4^) and the percentage of sialylation on non-fucosylated compositions being α2,3-linked (A2F0GRle; 9.2% *vs.* 6.5%; *p* = 2.4∙10^−5^).

### Triantennary Traits

The major changes observed in TPNG were H6N5E1L2 and H6N5E2L1 which both showed 4- to 5-fold lower levels in the UC plasma. The second largest difference is observed for the H6N5E1L1 glycan which showed 2.5-fold lower levels in the UC plasma. This group of triantennary non-fucosylated but sialylated glycans strongly influences many of the calculated derived traits that are listed below. A higher level of fucosylation of triantennary structures (A3F; 26.9% *vs.* 49.1%; *p* = 2.2∙10^−5^) was observed for UC. Alternatively, UC showed lower levels of sialylation (A3S; 92.6% *vs.* 87.2%; *p* = 4.2∙10^−6^), sialylation of non-fucosylated compositions (A3F0S; 91.7% *vs.* 83.2%; *p* = 2.4∙10^−5^), sialylation of fucosylated compositions (A3FS; 93.7% *vs.* 91.1%; *p* = 1.3∙10^−4^), α2,3-linked sialylation (A3L; 33.3% *vs.* 29.5%; *p* = 1.2∙10^−5^), α2,3-linked sialylation of non-fucosylated compositions (A3F0L; 30.4% *vs.* 22.7%; *p* = 3.0∙10^−5^), α2,3-linked sialylation of fucosylated compositions (A3FL; 40.6% *vs.* 36.3%; *p* = 3.0∙10^−5^), sialylation per galactose (A3GS; 92.6% *vs.* 87.2%; *p* = 4.2∙10^−6^), sialylation per galactose of non-fucosylated compositions (A3F0GS; 91.7% *vs.* 83.2%; *p* = 2.4∙10^−5^), sialylation per galactose of fucosylated compositions (A3FGS; 93.7% *vs.* 91.1%; *p* = 1.3∙10^−4^), α2,3-linked sialylation per galactose (A3GL; 33.3% *vs.* 29.5%; *p* = 1.2∙10^−5^), α2,3-linked sialylation per galactose of non-fucosylated compositions (A3F0GL; 30.4% *vs.* 22.7%; *p* = 3.0∙10^−5^), α2,3-linked sialylation per galactose of fucosylated compositions (A3FGL; 40.6% *vs.* 36.3%; *p* = 3.0∙10^−5^), the percentage of sialylation being α2,3-linked (A3GRle; 36.0% *vs.* 33.8%; *p* = 2.5∙10^−4^), the percentage of sialylation on non-fucosylated compositions being α2,3-linked (A3F0GRle; 33.0% *vs.* 27.2%; *p* = 3.9∙10^−5^) and the percentage of sialylation on fucosylated compositions being α2,3-linked (A3FGRle; 43.4% *vs.* 39.9%; *p* = 9.3∙10^−5^).

## Discussion

The humoral immune defence of newborns relies on supply of maternal IgG. IgG is the major plasma glycoprotein under normal circumstances, with concentrations ranging from 6.7 to 14.5 mg/mL in healthy females^3^. During pregnancy, FcRn is generally considered to be the sole receptor mediating the active transport of IgG across the placenta^25^. This is in line with observations that glycosylation of the IgG-Fc does not seem to affect transport, despite slight influence of IgG glycosylation on the *in vitro* binding to FcRn^8^,^26^. The net result is that the IgG found in the newborns, being almost exclusively maternal derived, closely resembles what is found at the maternal site^14^. However, not much is known regarding the differences in glycosylation between the maternal and UC plasma, the latter being commonly used to approximate foetal plasma^12^,^13^. Because of this, we applied a recently developed sialic acid derivatisation method to released *N*-glycans originating from both maternal and umbilical cord IgG and plasma, obtained after an average gestational age of 37.8 weeks^22^. The derivatised glycans were measured by MALDI-TOF-MS in order to identify differences in the *N*-glycomes.

For the analysis of total plasma as well as IgG *N*-glycome data, *N*-glycan values were normalised to the sum of intensities which allows more robust analysis. As a consequence of the total glycan area normalisation negative correlations are often observed (*e.g.* a higher level of diantennary composition will lead to a lower relative abundance of triantennary compositions). Furthermore, by analysing compositions of glycans released from their protein backbone, observed changes may arise from changes in (site-specific) glycosylation as well as changes in glycoprotein levels. These uncertainties can be mitigated to some degree by making use of glycosylation traits within 1 group of glycans (*e.g.* diantennary), and by using established literature regarding the most abundant plasma glycoproteins^24^. Our results clearly show a different glycosylation profile between maternal and UC plasma and to a lesser extent between maternal and UC IgG.

We confirmed that UC IgG has a higher level of galactosylation than maternal IgG, in line with previous reports^12^,^13^,^27^. Furthermore, we observed lower levels of bisection, sialylation and sialylation per galactose (Fig. 3). However, the effect size of all these changes was rather small, with the biggest change being about 2.5% in the sialylation per galactose. One explanation for observing only minor differences in IgG glycosylation between the mother and child is that the majority of foetal IgG is derived from the mother via an active transport mechanism that does not discriminate between IgG-Fc glycoforms^4^. The observed differences could be caused by a selective transport mechanism via the FcRn, although the method or mechanism behind this transport remains unknown^11^. By analysing IgG-derived Fc-glycopeptides we previously found no difference in the Fc glycosylation between maternal and UC IgG within each IgG subclass^14^. In contrast, the studies performed in this paper were performed at the released glycans level and clearly showed some – albeit minor – differences in glycosylation. These differences may be attributed to a transportation bias for IgG subclasses, which has been shown to have a transport preference (*e.g.* IgG1 > IgG2)^28^, or the preference for certain Fab-glycosylated IgG species. Specifically, the analysed IgG contained both the Fc and the Fab derived glycans, with the Fab glycans being found on ~20% of IgG in healthy adults and to this date there has been no investigation into Fab glycosylation in UC and the effect of Fab glycosylation on transport of IgG^29^. Alternatively, the difference could be caused by foetal IgG production, although the foetus has been shown to only produce minor levels of IgG, starting from the 20^th^ week of gestation^5^. Another potential cause for the difference is the higher protein age of the maternally produced IgGs when present in the foetus. After production in the mother the IgGs need to be transported to the foetus. While in the circulation the glycans on glycoproteins are believed to be slowly trimmed down, starting with the sialic acids^30^. Interestingly, we indeed do observe lower levels of IgG sialylation in the UC than in the maternal IgG (Table 1; A2GS; 23.3% *vs.* 26.1%), adding credibility to the glycoprotein ageing hypothesis, even though there currently is a lack of literature proof for the asialoglycoprotein receptor family (ASGPR) involvement with IgG recycling. A final possibility might be contamination of the IgG glycosylation with other non-IgG contaminants, potentially due to aspecific binding during the IgG purification step.

The TPNG comparison showed more pronounced differences between mother and UC than observed for IgG, the 2 most prominent differences being observed for the glycans H6N5E2L1 and H6N5E1L2, which both showed a 4 or 5-fold lower level in the UC. These triantennary trisialylated glycans are generally found on acute phase proteins such as alpha-1-acid glycoprotein and alpha-1-antitrypsin^24^. These glycans are of major influence on the observed fucosylation of triantennary glycans (A3F), *i.e.* 1.8-fold higher in the UC (27% in maternal and 49% in UC plasma), as well as on the lower UC levels of specifically α2,3-linked sialylation (A3S and A3L).

In all, the differences between mother and UC TPNG may be caused by different protein content, or different glycosylation of the maternal and UC plasma proteins. With respect to the first, there is literature on differential protein expression levels. For example, newborns have 1.65 ± 0.44 mg/mL of serotransferrin, while women have 3.23 ± 0.51 mg/mL just after delivery^31^. Another example is alpha-1-antitrypsin, which in newborns at term has a concentration of 3.0 mg/mL, while in mothers it has a concentration between 0.51 and 2.44 mg/mL^3^,^32^. Furthermore, there are proteins such as alpha-fetoprotein (AFP), which are predominantly present during foetal development^33^. This demonstrates that foetal plasma protein content is distinct from that of the mother, which could explain part of the observed differences. Alternatively, the difference could again be caused by a transport mechanism although this is unlikely because no study to date has found any active transport of proteins other than IgG from the mother to the foetus^10^,^34^,^35^. However, inflammatory conditions may induce translocation of for example IgA and IgM, although these immunoglobulins possess mainly diantennary and some oligomannosidic glycans and could therefore only account for a limited number of the observed differences between mother and UC, and the quantity as well as the diversity of glycosylation of the translocation may be limited^24^,^36^.

Lastly, another hypothesis may be that reduced clearance via the ASGPR could contribute to the low levels of sialylated glycans in the UC. Under normal circumstances these receptors clear glycoproteins by binding to the glycans lacking a terminal sialic acid^37^. Furthermore, the ASGPR clears glycoproteins with a terminal α2,6-linked sialic, albeit to a far lower rate than glycans with a terminal galactose^38^,^39^. Reduced clearance via the ASGPR would lead to a relative decrease in sialylation, and more specifically a shift from terminal α2,3-linked to α2,6-linked sialic acids, which matches well with our results. Clearly, additional work is needed to reveal foetal glycoprotein production and turnover. Additionally, placental transport maybe investigated making use of potential allotype differences between foetus and mother, specifically identifying maternal allotype in the foetal circulation that can serve as an indication of transport. Furthermore, the usage of IgG-depleted sera can be used to ensure that no IgG derived effects cause false positive results in the analysis of other plasma proteins.

Interestingly, while most effects observed for IgG were also detectable in TPNG, this was not the case for galactosylation. IgG shows higher UC levels of galactosylation (A2G), whereas in the TPNG no changes in galactosylation of fucosylated diantennary compositions (A2FG) were observed. The majority of these fucosylated diantennary glycans are believed to originate from IgG^24^. This suggests that other glycoproteins that carry fucosylated diantennary compositions, such as IgM and IgA, may contribute with low galactosylation to the UC TPNG profile, thereby counteracting IgG with its elevated galactosylation in UC. However, while in normal healthy people IgM, IgG and IgA are the main contributors to the group of fucosylated diantennary compositions, in newborns the levels of IgM (641.3 μg/mL) and IgA (54.1 μg/mL) are significantly lower relative to IgG (8.47 mg/mL at 38 weeks), implying that different proteins might be involved^40^,^41^.

In conclusion, this study shows that maternal and UC TPNG and, to a lesser degree, maternal and UC IgG differ in their glycosylation. The cause for the non-IgG related differences remains unclear and will require further study towards potential active transport of other proteins, protein half-life in foetal plasma, and protein clearance. For example, profiling purified proteins from total plasma will give information regarding protein-specific glycosylation differences. Furthermore, measuring glycopeptides instead of released *N*-linked glycans will provide additional information regarding site-specific glycosylation differences.

## Materials and Methods

### Patient samples

Plasma was collected from the 42 mothers and the umbilical cord of their newborns directly after delivery (average 37.8 weeks). All pregnancies were without complications and all children had optimal neonatal outcomes, meaning no complications at full term. Mothers gave signed informed consent prior to the study. The collection of plasma samples and clinical data was in compliance with the Helsinki declaration and was approved by the Ethics Committee of the Leiden University Medical Center (P02-200). Furthermore, all methods and experiments were performed in accordance with the relevant ethical guidelines and regulations.

### Chemicals, Reagents and Enzymes

Milli-Q water (MQ) was taken from a Q-Gard 2 system (Millipore, Amsterdam, Netherlands), maintained at ≥18 MΩ. Ethanol, sodium dodecyl sulphate (SDS), disodium hydrogen phosphate dihydrate (Na_2_HPO_4_∙2H_2_O; ≥99.5% purity), potassium dihydrogen phosphate (KH_2_PO_4_; ≥99.5% purity) were purchased from Merck (Darmstadt, Germany). 1-Hydroxybenzotriazole (HOBt; ≥97.0% purity), tosyl phenylalanyl chloromethyl ketone (TPCK)-treated trypsin, Nonidet P-40 substitute (NP-40) and hydrochloric acid (HCl) were purchased from Sigma-Aldrich (Steinheim, Germany). 1-Ethyl-3-(3-dimethylaminopropyl)carbodiimide (EDC; 99.0% purity) was acquired from Fluorochem (Hadfield, UK). Peptide-*N*-glycosidase F (PNGase F) was acquired from Roche Diagnostics (Mannheim, Germany). Super-DHB (9:1 mixture of 2,5-dihydroxybenzoic acid and 2-hydroxy-5-methoxybenzoic acid), peptide calibration standard and MTP AnchorChip 800/384 MALDI target plates originated from Bruker Daltonics (Bremen, Germany). HPLC SupraGradient acetonitrile (ACN; ≥99.97 purity) was acquired from Biosolve (Valkenswaard, Netherlands). CaptureSelect^TM^ IgG-Fc (Hu) beads were bought from ThermoFisher Scientific (Waltham, USA). The AcroPrep^TM^ 96-well filter plates with a GHP membrane originated from Pall Corporation (Ann Arbor, USA). Phosphate-buffered saline (PBS) was made in-house, containing 5.7 g/L Na_2_HPO_4_∙2H_2_O, 0.5 g/L KH_2_PO_4_ and 8.5 g/L NaCl. A pool of healthy control plasma was acquired from Affinity Biologicals (Ancaster, Canada). Filter plates (10 μm pore size) were purchased from Orochem (Naperville, IL, USA).

### IgG Capturing

IgG was affinity captured from plasma using IgG Fc beads. 20 μL of IgG Fc bead slurry was transferred to each well of a 96-wells Orochem filter plate. All wells were washed 3 times with 200 μL PBS, using a vacuum manifold. 5 μL of plasma was incubated on the Fc beads together with 100 μL PBS for 1 h on a shaker (1050 rpm). Subsequently, the filter plates were washed 3 times with 200 μL PBS. Purified IgG was collected with an elution step using 100 μL 100 mM HCl into 20 μL 500 mM NaOH. 40 μL of the 120 μL eluate was transferred to a V-bottom plate and dried using a vacuum centrifuge.

### Glycan Release & Ethyl Esterification

IgG glycans were released by adding 10 μL of PBS to the dried purified IgG protein. Additionally, 20 μL of 2% SDS was added to the samples prior to a 30 min incubation at 60 °C. Afterwards, 20 μL of release mixture (1:1 4% NP-40:5× PBS & 0.25 units of PNGase F) was added to each well and the mixture was incubated overnight at 37 °C. Alternatively, the total plasma glycans were released by adding 6 μL of 1× PBS to 6 μL mother/UC or control plasma. Additionally, 12 μL of 2% SDS was added to the samples prior to a 10 min incubation at 60 °C. After incubation 12 μL of release mixture (1:1 4% NP-40:5× PBS & 0.75 units of PNGase F) was added to the samples and incubated over night at 37 °C.

The released IgG glycans were manually ethyl esterified by adding 100 μL of EDC/HOBt (0.5 M in EtOH) to 20 μL of released glycans prior to a 1 h incubation step at 37 °C. Alternatively, released TPNG glycans were ethyl esterified using a previously described robot protocol^42^. Briefly, 60 μL of ethyl esterification reagent, consisting of 250 mM EDC and 250 mM HOBt in ethanol, was added to each well. The robot then added 3 μL of released TPNG glycans to each well, prior to a 75 min incubation at room temperature. Lastly, 120 μL of ACN was added to each well as a preparation for hydrophilic interaction liquid chromatography (HILIC) purification.

### Glycan Purification

Ethyl esterified IgG glycans were manually purified using a cotton HILIC solid phase extraction (SPE) as described previously^43^. Briefly, the cotton material was pre-conditioned by pipetting 3 times 20 μL MQ, followed by equilibration with 3 times 20 μL 85% ACN. Ethyl esterified IgG glycans were loaded onto the cotton material by repeated pipetting of the release mixture. The tips were sequentially washed with 3 times 20 μL 85% ACN + 1% TFA and 3 times 85% ACN. The purified ethyl esterified IgG glycans were eluted in 30 μL water.

Alternatively, the ethyl esterified TPNG glycans were purified using a previously published robot protocol^42^. Briefly, a 96-well filter plate with a GHP membrane was pre-wetted, activated and equilibrated using 100 μL of 70% ethanol, 100 μL water and 100 μL ACN, respectively. Afterwards, the reaction mixture (183 μL) was loaded onto the GHP plate and incubated for 5 min, after which a pressure gradient of about 3 kPa was applied to the plate. The GHP plate was washed 3 times using 100 μL of 96% ACN, and residual solvents were removed by gently tapping the bottom of the plate on a disposable hand towel. The resulting purified ethyl esterified TPNG glycans were subsequently eluted by adding 30 μL of water, followed by 5 min incubation at room temperature. The final recovery was achieved by centrifuging the plate for 5 min at roughly 300× *g*.

### MALDI-TOF-MS

Ethyl esterified IgG and TPNG glycans were spotted on a MTP AnchorChip 800/384 MALDI target plate by mixing 5 μL of the SPE eluate with 1 μL of 5 mg/mL Super-DHB in 50% aqueous ACN containing 1 mM NaOH, prior to drying on the MALDI target plate. An Ultraflextreme MALDI-TOF-MS (Bruker Daltonics), equipped with Smartbeam-II laser was used to measure the ethyl esterified glycan samples in reflectron positive ion mode. The MALDI-TOF-MS was operated by flexControl 3.4, build 135. The MALDI-TOF-MS was calibrated using a peptide calibration standard prior to measurement. An acceleration voltage of 25 kV was applied following a 140 ns extraction delay. The laser power was set as high as possible while still resulting in isotopic resolution. A total of 20,000 shots at 1,000 Hz were accumulated per spectrum with a mass range of *m/z* 1,000 to *m/z* 5,000.

### MassyTools Processing

All spectra were converted into “.xy” files using Bruker flexAnalysis, prior to processing with MassyTools^23^. Briefly, MassyTools calibrated each spectrum based on a list of glycan compositions (Supplementary Tables S1 and S3). Subsequently, MassyTools performed targeted extraction, relative quantification, and quality criterion calculation on basis of a second set of glycan compositions (Supplementary Tables S1 and S3).

For MassyTools processing, we applied the ‘free’ and ‘sodium’ mass modifiers to all compositions, reflecting the conditions of our experiments. Compositions were built using the standard MassyTools building blocks of hexose (H), *N-*acetylhexosamine (N), fucose (F), ethyl esterified *N*-acetylneuraminic acid (E) and lactonised *N*-acetylneuraminic acid (L). All IgG samples were calibrated using MassyTools, using at least 4 internal calibrants of which 2 had to be either H3N4F1, H4N4F1 or H5N4F1 and 2 had to be either H5N4F1E1, H5N4F1E2 or H5N5F1E2. All TPNG samples were calibrated using at least 5 internal calibrants, of which at least 3 had to be either H3N4F1, H4N4F1, H5N4F1, H5N4E1 or H5N4F1E1 and 1 had to be either H6N5E2L1, H6N5F1E2L1 or H7N6E1L3. For quantitation, we used 95% of the theoretical isotopic pattern, where a window of ±0.49 Th (Da/e) was integrated around each isotope and the program was allowed to look for the local background and noise in a window of ±20 Th around the monoisotopic peak.

### Spectrum & Analyte Selection

A sum spectrum was created for each category, *i.e.* maternal TPNG, UC TPNG, maternal IgG and UC IgG, using all spectra that passed the calibration step for each category. Glycan compositions were manually identified from these sum spectra, any potential composition that was present in at least 1 sum spectrum would be extracted from the relevant group. A total of 45 potential IgG compositions and 109 TPNG compositions were selected for quantitation. All samples that passed calibration and could be matched to their respective maternal or UC partner, meaning that both spectra of a given pair passed calibration, were used for the quantitation. This resulted in 4 pairs of IgG data and 10 pairs of TPNG data being removed, leaving 38 pairs of IgG data and 32 pairs of TPNG data for further quantitation. Initial quantitation of the TPNG and IgG data was performed using a list of previously identified glycans, as no novel glycans were identified in sum spectra of the maternal or UC profiles. Subsequently, analytes were curated using the isotopic pattern quality (IPQ), signal-to-noise ratio (S/N) and the mass accuracy as reported by MassyTools^23^. Analytes were only retained if in either the maternal or UC origin the following criteria were fulfilled: an average mass error between −20 and +20 ppm (Supplementary Tables S5 and S6), an average S/N above 3, and the average IPQ had to pass the IPQ cut-off. The IPQ cut-off was dynamic, specifically for the most abundant analyte the IPQ had to be below 5% while for the least abundant analyte it had to be below 25%. For example, with X analytes the 3^rd^ most abundant analyte had to have an IPQ below 5% + ((25-5)/X)*3), which would be 6.33% when X is 45. However, as curation is an iterative process, we recalculated the IPQ cut-off after every curated analyte and examined all analytes if they passed the selected criteria. Utilizing these criteria we curated 64 TPNG analytes, retaining 45. For the IgG samples we curated 8 analytes, retaining 37. These individual glycans were grouped into derived glycosylation traits (Supplementary Table S2).

### Statistical Analysis

Individual glycans and derived traits were tested pairwise using a Wilcoxon signed-rank test, since several traits were not normally distributed^44^. We used a significance threshold of α = 0.05 before multiple testing correction, which was performed using a Bonferroni correction^45^. We treated each set of results as a separate dataset, *i.e.* we corrected for 37 tests in the case of the IgG glycans and thus used a significance threshold of α = 0.0014. A threshold of α = 0.0071 was used for the IgG derived traits, α = 0.00011 for the TPNG glycans and α = 0.000735 for the TPNG derived traits.

### Software

We used GlycoWorkbench 2 for the glycan cartoons^46^. The conversion of raw MS data to an “.xy” file format was achieved using the flexAnalysis BatchProcess program. All statistical analyses were performed using IBM SPSS 20. Tables and pictures were formatted using Microsoft Office 2010. Lastly, the MALDI-TOF-MS was controlled using Bruker flexControl 3.4, build 135 (Bremen, Germany).

## Additional Information

**How to cite this article**: Jansen, B. C. *et al*. MALDI-TOF-MS reveals differential *N*-linked plasma- and IgG-glycosylation profiles between mothers and their newborns. *Sci. Rep.* **6**, 34001; doi: 10.1038/srep34001 (2016).

## Supplementary Material

Supplementary Information

## Figures and Tables

**Table 1:** Derived trait comparison between maternal and UC IgG.

Trait	Maternal	UC	Direction	Fold	*p*-value
M	1.26%	1.21%	—	—	0.62650
A2B	16.74%	15.60%	↓	0.93	**0.00004**
A2F	90.25%	90.33%	—	—	0.60667
A2G	72.95%	75.18%	↑	1.03	<**0.00001**
A2S	18.99%	17.52%	↓	0.92	**0.00205**
A2GS	26.05%	23.27%	↓	0.89	**0.00005**
TaGal	9.83%	7.75%	↓	0.79	**0.00000**

The relative abundance of immunoglobulin G (IgG) derived glycosylation traits is shown, together with the direction and fold change if the derived trait shows a significant difference between maternal and UC IgG (α = 0.0071; 7 tests) and their *p*-values are in bold. A single letter notation is used in this table with M referring to mannose, A to antenna, F to fucose, G to galactose and S to *N*-acetylgalactosamine. For a full description of the derived traits and their calculation refer to Supplementary Table S2.

**Table 2:** TPNG derived glycosylation trait comparison between maternal and UC plasma.

Trait	Maternal	UC	Direction	Fold	*p*-value
M	6.68%	5.79%	—	—	0.47736
Hy	0.55%	0.87%	↑	1.59	**0.00054**
C	92.11%	92.62%	—	—	0.88109
A1	0.65%	0.72%	—	—	0.26189
A2	77.09%	85.14%	↑	1.10	**0.00001**
A3	14.87%	7.34%	↓	0.49	<**0.00001**
B	5.94%	5.91%	—	—	0.80794
F	31.08%	39.72%	↑	1.28	**0.00001**
MM	7.30	7.29	—	—	0.70842
AG	94.75%	92.85%	↓	0.98	**0.00011**
AS	75.76%	65.45%	↓	0.86	**0.00001**
AL	12.01%	7.89%	↓	0.66	**0.00001**
AE	63.75%	57.56%	↓	0.90	**0.00005**
GS	79.73%	70.35%	↓	0.88	**0.00001**
GL	12.43%	8.35%	↓	0.67	**0.00001**
GE	67.30%	61.99%	↓	0.92	**0.00006**
AGRle	15.58%	11.84%	↓	0.76	**0.00001**
IGG	11.93%	20.12%	↑	1.69	**0.00001**
Diantennary Traits					
A2F	34.24%	41.38%	↑	1.21	**0.00003**
A2B	7.72%	6.98%	—	—	0.01041
A2F0B	0.88%	0.77%	—	—	0.26992
A2FB	21.03%	15.84%	↓	0.75	**0.00007**
A2G	93.77%	92.25%	—	—	0.00122
A2F0G	97.77%	97.44%	—	—	0.12520
A2FG	86.60%	84.97%	—	—	0.00448
A2S	72.58%	63.58%	↓	0.88	**0.00003**
A2F0S	89.24%	87.57%	—	—	0.01099
A2FS	41.49%	29.79%	↓	0.72	**0.00001**
A2L	7.89%	6.01%	↓	0.76	**0.00001**
A2F0L	8.17%	5.69%	↓	0.70	**0.00003**
A2FL	7.72%	6.54%	—	—	0.00633
A2E	64.69%	57.57%	↓	0.89	**0.00006**
A2F0E	81.07%	81.88%	—	—	0.41065
A2FE	33.77%	23.24%	↓	0.69	**0.00003**
A2GS	77.30%	68.90%	↓	0.89	**0.00002**
A2F0GS	91.27%	89.87%	—	—	0.00295
A2FGS	47.73%	35.01%	↓	0.73	**0.00001**
A2GL	8.40%	6.51%	↓	0.78	**0.00001**
A2F0GL	8.36%	5.85%	↓	0.70	**0.00003**
A2FGL	8.84%	7.69%	—	—	0.01099
A2GE	68.90%	62.39%	↓	0.91	**0.00004**
A2F0GE	82.91%	84.02%	—	—	0.07880
A2FGE	38.89%	27.33%	↓	0.70	**0.00003**
A2GRle	10.88%	9.45%	↓	0.87	**0.00011**
A2F0GRle	9.16%	6.51%	↓	0.71	**0.00002**
A2FGRle	18.41%	21.87%	↑	1.19	**0.00051**
Triantennary Traits					
A3F	26.86%	49.06%	↑	1.83	**0.00002**
A3G	100.00%	100.00%	—	—	*
A3F0G	100.00%	100.00%	—	—	*
A3FG	100.00%	100.00%	—	—	*
A3S	92.56%	87.24%	↓	0.94	<**0.00001**
A3F0S	91.68%	83.19%	↓	0.91	**0.00002**
A3FS	93.65%	91.06%	↓	0.97	**0.00013**
A3L	33.30%	29.51%	↓	0.89	**0.00001**
A3F0L	30.36%	22.67%	↓	0.75	**0.00003**
A3FL	40.61%	36.29%	↓	0.89	**0.00003**
A3E	59.26%	57.74%	—	—	0.00475
A3F0E	61.32%	60.52%	—	—	0.11625
A3FE	53.04%	54.76%	—	—	0.03623
A3GS	92.56%	87.24%	↓	0.94	<**0.00001**
A3F0GS	91.68%	83.19%	↓	0.91	**0.00002**
A3FGS	93.65%	91.06%	↓	0.97	**0.00013**
A3GL	33.30%	29.51%	↓	0.89	**0.00001**
A3F0GL	30.36%	22.67%	↓	0.75	**0.00003**
A3FGL	40.61%	36.29%	↓	0.89	**0.00003**
A3GE	59.26%	57.74%	—	—	0.00475
A3F0GE	61.32%	60.52%	—	—	0.11625
A3FGE	53.04%	54.76%	—	—	0.03623
A3GRle	35.96%	33.82%	↓	0.94	**0.00025**
A3F0GRle	33.02%	27.19%	↓	0.82	**0.00004**
A3FGRle	43.36%	39.86%	↓	0.92	**0.00009**

The mean value of all derived TPNG glycosylation traits is displayed for both maternal and UC origin, the direction and fold change is also displayed for significant (α = 0.000735; 68 tests) traits and their *p*-values are bolded. Traits that showed identical values for both maternal and UC plasma were not tested for significance (marked with a * in the *p*-value column). This table uses a single letter notation, with M referring to mannose, F to fucose, G to galactose, A to antenna, C to complex compositions, Hy to hybrid compositions, B to bisection, S to *N*-acetylneuraminic acid, E to ethyl esterified (α2,6-linked) *N*-acetylneuraminic acid, L to lactonised (α2,3-linked) *N*-acetylneuraminic acid and RLe refers to the ratio of E/(E + L). For a full description of the derived traits and their calculation refer to Supplementary Table S4.
