# Supplementary material for: MALDI-TOF-MS reveals differential N-linked plasma- and IgG-glycosylation profiles between mothers and their newborns
Source: Sci Rep. 2016 Sep 26;6:34001. doi: 10.1038/srep34001 (PMC5036037; doi:10.1038/srep34001)
Supplement: Supplementary Information [file srep34001-s1.pdf]

Supplementary Information for “MALDI-TOF-MS reveals differential *N*-linked plasma- and IgG-glycosylation profiles between mothers and their newborns”

Bas C. Jansen[1], Albert Bondt[1,2], Karli R. Reiding[1], Sicco A. Scherjon[3], Gestur Vidarsson[4] and Manfred Wuhrer[1,*]

[1]Center for Proteomics and Metabolomics, Leiden University Medical Center, 2300 RC Leiden, The Netherlands.
[2]Department of Rheumatology, Leiden University Medical Center, 2300 RC Leiden, The Netherlands.
[3]Department of Obstetrics and Gynaecology, University Medical Center Groningen, 9713 GZ Groningen, The Netherlands.
[4]Department of Experimental Immunohematology, Sanquin Research, Amsterdam, The Netherlands.

[*]Corresponding author: Manfred Wuhrer, m.wuhrer@lumc.nl, Tel. +31-71-52-68744

Table of Contents

Supplemental Table S1: Released glycan comparison between maternal and UC IgG
Supplemental Table S2: IgG derived trait description
Supplemental Table S3: TPNG glycan comparison between maternal and UC plasma
Supplemental Table S4: TPNG derived trait description
Supplemental Table S5: IgG glycan accurate masses
Supplemental Table S6: TPNG glycan accurate masses

Supplemental Table S1: Released glycan comparison between maternal and UC IgG. The relative abundance of immunoglobulin G (IgG) derived glycans is shown, together with the direction and fold change if the glycan shows a significant difference between maternal and umilical cord derived from newborn children (UC) IgG (significance threshold α = 0.0014; 37 tests) and their p-values are bolded. Furthermore, glycans that did not pass curation are also presented separated into either non quantified or non detected glycans. The number of spectra in which a non quantified glycan passed the curation limit is presented. Asterisks denote glycans that were used for calibration.

Glycan	Maternal	UC	Direction	Fold	*p* -value
H5N2	0.28%	0.34%	-	-	1.0000
H3N4	0.81%	0.58%	-	-	0.6265
H6N2	0.15%	0.14%	-	-	0.8711
H3N4F1*	6.26%	4.79%	↓	0.76	**< 0.0001**
H4N4	1.22%	1.30%	-	-	0.0150
H3N5	0.37%	0.35%	-	-	0.2561
H7N2	0.14%	0.13%	-	-	0.4173
H4N4F1*	23.33%	22.95%	-	-	0.8711
H5N4	2.00%	2.35%	↑	1.18	**0.0001**
H3N5F1	2.26%	1.94%	↓	0.86	**< 0.0001**
H4N5	0.49%	0.51%	-	-	0.6265
H8N2	0.39%	0.36%	-	-	0.2561
H4N4L1	0.13%	0.13%	-	-	0.4173
H5N4F1*	23.24%	25.83%	↑	1.11	**< 0.0001**
H4N4E1	0.32%	0.35%	-	-	0.8711
H4N5F1	5.59%	6.07%	↑	1.09	**< 0.0001**
H5N5	0.27%	0.28%	-	-	0.6265
H9N2	0.31%	0.25%	-	-	0.6265
H5N4L1	0.17%	0.15%	-	-	1.0000
H4N4F1E1	2.36%	1.91%	↓	0.81	**< 0.0001**
H5N4E1	1.48%	1.57%	-	-	0.6265
H5N5F1	1.90%	2.03%	-	-	0.0350
H4N5E1	0.16%	0.16%	-	-	0.4173
H5N4F1L1	0.32%	0.35%	-	-	0.0150
H5N4F1E1*	16.39%	17.90%	↑	1.09	**0.0001**
H4N5F1E1	0.41%	0.34%	-	-	0.0021
H5N5E1	0.50%	0.48%	-	-	0.8711
H5N4E1L1	0.28%	0.22%	-	-	0.0350
H5N5F1L1	0.13%	0.13%	-	-	0.6265
H5N4E2	1.13%	0.90%	-	-	0.0021
H5N5F1E1	2.15%	1.52%	↓	0.71	**< 0.0001**
H5N4F1E1L1	0.18%	0.15%	-	-	**0.0350**
H5N4F1E2*	2.59%	1.95%	↓	0.75	**< 0.0001**
H5N5E2	0.28%	0.21%	-	-	0.0058
H5N5F1L2	0.09%	0.08%	-	-	0.8711
H5N5F1E1L1	0.11%	0.09%	-	-	0.0350
H5N5F1E2*	1.81%	1.20%	↓	0.67	**< 0.0001**
Non quantified	Maternal	UC	Direction	Fold	*p* -value
H5N5L2	4 [38]	3 [38]	-	-	-
H5N5E1L1	3 [38]	3 [38]	-	-	-
Non detected	Maternal	UC	Direction	Fold	*p* -value
H7N6	-	-	-	-	-
H7N6F1	-	-	-	-	-
H7N7	-	-	-	-	-
H7N7F1	-	-	-	-	-
H7N6F1E1	-	-	-	-	-
H7N6F1E2	-	-	-	-	-

Supplemental Table S2: IgG Derived trait description. This table describes the calculation of each derived trait of IgG, lists a simple description and a graphical representation of the minimal glycan structure for each derived trait. The curly bracket in the graphical representation is used to indicate the part of the glycan that is represented by the derived trait, *e.g.* the galactose on the A2G trait indicates that it is the percentage of galactosylation that is represented.

Trait name	Description	Minimal glycan structure	Calculation
M	% High mannose compositions	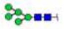	(1 * (H5N2 + H6N2 + H7N2 + H8N2 + H9N2)) / (1 * (H5N2 + H3N4 + H6N2 + H3N4F1 + H4N4 + H3N5 + H7N2 + H4N4F1 + H5N4 + H3N5F1 + H4N5 + H8N2 + H4N4L1 + H5N4F1 + H4N4E1 + H4N5F1 + H5N5 + H9N2 + H5N4L1 + H4N4F1E1 + H5N4E1 + H5N5F1 + H4N5E1 + H5N4F1L1 + H5N4F1E1 + H4N5F1E1 + H5N5E1 + H5N4E1L1 + H5N5F1L1 + H5N4E2 + H5N5F1E1 + H5N4F1E1L1 + H5N4F1E2 + H5N5E2 + H5N5F1L2 + H5N5F1E1L1 + H5N5F1E2))
A2B	% Bisection of diantennary compositions	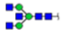	(1 * (H3N5 + H3N5F1 + H4N5 + H4N5F1 + H5N5 + H5N5F1 + H4N5E1 + H4N5F1E1 + H5N5E1 + H5N5F1L1 + H5N5F1E1 + H5N5E2 + H5N5F1L2 + H5N5F1E1L1 + H5N5F1E2)) / (1 * (H5N2 + H3N4 + H6N2 + H3N4F1 + H4N4 + H3N5 + H7N2 + H4N4F1 + H5N4 + H3N5F1 + H4N5 + H8N2 + H4N4L1 + H5N4F1 + H4N4E1 + H4N5F1 + H5N5 + H9N2 + H5N4L1 + H4N4F1E1 + H5N4E1 + H5N5F1 + H4N5E1 + H5N4F1L1 + H5N4F1E1 + H4N5F1E1 + H5N5E1 + H5N4E1L1 + H5N5F1L1 + H5N4E2 + H5N5F1E1 + H5N4F1E1L1 + H5N4F1E2 + H5N5E2 + H5N5F1L2 + H5N5F1E1L1 + H5N5F1E2))
A2F	% Fucosylation of diantennary compositions		(1 * (H3N4F1 + H4N4F1 + H3N5F1 + H5N4F1 + H4N5F1 + H4N4F1E1 + H5N5F1 + H5N4F1L1 + H5N4F1E1 + H4N5F1E1 + H5N5F1L1 + H5N5F1E1 + H5N4F1E1L1 + H5N4F1E2 + H5N5F1L2 + H5N5F1E1L1 + H5N5F1E2)) / (1 * (H5N2 + H3N4 + H6N2 + H3N4F1 + H4N4 + H3N5 + H7N2 + H4N4F1 + H5N4 + H3N5F1 + H4N5 + H8N2 + H4N4L1 + H5N4F1 + H4N4E1 + H4N5F1 + H5N5 + H9N2 + H5N4L1 + H4N4F1E1 + H5N4E1 + H5N5F1 + H4N5E1 + H5N4F1L1 + H5N4F1E1 + H4N5F1E1 + H5N5E1 + H5N4E1L1 + H5N5F1L1 + H5N4E2 + H5N5F1E1 + H5N4F1E1L1 + H5N4F1E2 + H5N5E2 + H5N5F1L2 + H5N5F1E1L1 + H5N5F1E2))
A2G	% Galactosylation of diantennary compositions	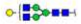	(1/2 * (H4N4 + H4N4F1 + H4N5 + H4N4L1 + H4N4E1 + H4N5F1 + H4N4F1E1 + H4N5E1 + H4N5F1E1) + 2/2 * (H5N4 + H5N4F1 + H5N5 + H5N4L1 + H5N4E1 + H5N5F1 + H5N4F1L1 + H5N4F1E1 + H5N5E1 + H5N4E1L1 + H5N5F1L1 + H5N4E2 + H5N5F1E1 + H5N4F1E1L1 + H5N4F1E2 + H5N5E2 + H5N5F1L2 + H5N5F1E1L1 + H5N5F1E2)) / (1 * (H3N4 + H3N4F1 + H4N4 + H3N5 + H4N4F1 + H5N4 + H3N5F1 + H4N5 + H4N4L1 + H5N4F1 + H4N4E1 + H4N5F1 + H5N5 + H5N4L1 + H4N4F1E1 + H5N4E1 + H5N5F1 + H4N5E1 + H5N4F1L1 + H5N4F1E1 + H4N5F1E1 + H5N5E1 + H5N4E1L1 + H5N5F1L1 + H5N4E2 + H5N5F1E1 + H5N4F1E1L1 + H5N4F1E2 + H5N5E2 + H5N5F1L2 + H5N5F1E1L1 + H5N5F1E2))
A2S	% Sialylation of diantennary compositions	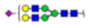	(1/2 * (H4N4L1 + H4N4E1 + H5N4L1 + H4N4F1E1 + H5N4E1 + H4N5E1 + H5N4F1L1 + H5N4F1E1 + H4N5F1E1 + H5N5E1 + H5N5F1L1 + H5N5F1E1) + 2/2 * (H5N4E1L1 + H5N4E2 + H5N4F1E1L1 + H5N4F1E2 + H5N5E2 + H5N5F1L2 + H5N5F1E1L1 + H5N5F1E2)) / (1 * (H3N4 + H3N4F1 + H4N4 + H3N5 + H4N4F1 + H5N4 + H3N5F1 + H4N5 + H4N4L1 + H5N4F1 + H4N4E1 + H4N5F1 + H5N5 + H5N4L1 + H4N4F1E1 + H5N4E1 + H5N5F1 + H4N5E1 + H5N4F1L1 + H5N4F1E1 + H4N5F1E1 + H5N5E1 + H5N4E1L1 + H5N5F1L1 + H5N4E2 + H5N5F1E1 + H5N4F1E1L1 + H5N4F1E2 + H5N5E2 + H5N5F1L2 + H5N5F1E1L1 + H5N5F1E2))
A2GS	% Sialylation per galactose of diantennary compositions	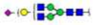	(1 * (A2S)) / (1 * (A2G))
TaGal	% Agalactosylated compositions of diantennary compositions	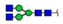	(H3N4 + H3N4F1 + H3N5 + H3N5F1) / (1 * (H3N4 + H3N4F1 + H4N4 + H3N5 + H4N4F1 + H5N4 + H3N5F1 + H4N5 + H4N4L1 + H5N4F1 + H4N4E1 + H4N5F1 + H5N5 + H5N4L1 + H4N4F1E1 + H5N4E1 + H5N5F1 + H4N5E1 + H5N4F1L1 + H5N4F1E1 + H4N5F1E1 + H5N5E1 + H5N4E1L1 + H5N5F1L1 + H5N4E2 + H5N5F1E1 + H5N4F1E1L1 + H5N4F1E2 + H5N5E2 + H5N5F1L2 + H5N5F1E1L1 + H5N5F1E2))

Supplemental Table S3: TPNG glycan comparison between maternal and UC plasma. The table displays the mean value of all individual total plasma *N*-glycome (TPNG) glycans, for glycans that show a significant change (α = 0.0011; 45 tests) the direction of the change and the fold change (UC/Maternal) are displayed and their p-values are bolded. Furthermore, glycans that did not pass curation are also presented separated into either non quantified or non detected glycans. The number of spectra in which a non quantified glycan passed the curation limit is presented. Asterisks denote glycans that were used for calibration.

Glycan	Maternal	UC	Direction	Fold	*p*-value
H5N2	0.35%	0.32%	-	-	0.42137
H3N4	0.34%	0.42%	-	-	0.05181
H6N2	2.03%	1.84%	-	-	0.79349
*H3N4F1**	0.86%	1.01%	-	-	0.00475
H4N4	0.69%	0.94%	-	-	0.00987
H7N2	1.15%	1.02%	-	-	0.72238
*H4N4F1**	3.53%	5.57%	↑	1.58	**0.00002**
H5N4	0.46%	0.82%	↑	1.79	**0.00004**
H3N5F1	0.27%	0.35%	↑	1.32	**0.00087**
H4N5	0.30%	0.25%	-	-	0.17820
H8N2	1.44%	1.05%	-	-	0.02041
H4N3F1E1	0.65%	0.72%	-	-	0.26189
H5N3E1	0.55%	0.87%	↑	1.59	**0.00054**
*H5N4F1**	5.29%	10.01%	↑	1.89	**0.00001**
H4N4E1	0.50%	0.46%	-	-	0.51281
H4N5F1	0.92%	1.48%	↑	1.61	**0.00001**
H9N2	1.71%	1.56%	-	-	0.79349
H4N4F1E1	0.80%	0.96%	-	-	0.00792
*H5N4E1**	6.63%	7.00%	-	-	0.41065
H5N5F1	1.07%	1.70%	↑	1.59	**0.00003**
H5N4L1F1	0.50%	0.63%	↑	1.26	**0.00031**
*H5N4F1E1**	4.47%	6.68%	↑	1.49	**< 0.00001**
H5N4L2	0.95%	0.68%	↓	0.72	**0.00009**
H5N4E1L1	6.33%	4.28%	↓	0.68	**0.00002**
H5N4E2	34.31%	34.86%	-	-	0.89586
H5N5F1E1	2.01%	1.32%	↓	0.66	**0.00015**
H6N5E1	0.58%	0.53%	-	-	0.22420
H5N4F1L2	1.07%	0.89%	-	-	0.00333
H4N6F1E1	0.14%	0.14%	-	-	0.26189
H5N4F1E1L1	1.23%	2.11%	↑	1.71	**0.00005**
H5N4F1E2	3.21%	1.91%	↓	0.60	**0.00015**
H6N5F1E1	0.22%	0.31%	-	-	0.00191
H5N5E2	0.13%	0.13%	-	-	0.94038
H6N5L2	0.23%	0.13%	-	-	0.00475
H6N5E1L1	0.81%	0.33%	↓	0.41	**0.00001**
H5N5F1E2	1.23%	0.67%	↓	0.55	**0.00006**
H6N5E2	0.35%	0.22%	↓	0.65	**0.00025**
H6N5F1E1L1	0.27%	0.29%	-	-	0.21027
H6N5E1L2	1.45%	0.34%	↓	0.24	**0.00001**
*H6N5E2L1**	6.15%	1.41%	↓	0.23	**0.00001**
H6N5E3	1.49%	0.79%	↓	0.53	**0.00001**
H6N5F1L3	0.39%	0.20%	↓	0.52	**0.00004**
H6N5F1E1L2	0.43%	0.36%	-	-	0.05895
*H6N5F1E2L1**	2.33%	2.27%	-	-	0.51281
H6N5F1E3	0.19%	0.15%	-	-	0.00935
Non quantified	Maternal	UC	Direction	Fold	*p*-value
H3N3	0 [32]	2 [32]	-	-	-
H3N3F1	1 [32]	0 [32]	-	-	-
H4N3	4 [32]	3 [32]	-	-	-
H4N3F1	3 [32]	3 [32]	-	-	-
H4N3L1	1 [32]	5 [32]	-	-	-
H4N3E1	1 [32]	2 [32]	-	-	-
H4N4L1	3 [32]	1 [32]	-	-	-
H5N5	1 [32]	0 [32]	-	-	-
H6N5F1L2	2 [32]	0 [32]	-	-	-

Non detected	Maternal	UC	Direction	Fold	*p*-value
H3N3L1	-	-	-	-	-
H3N3E1	-	-	-	-	-
H5N3	-	-	-	-	-
H3N5	-	-	-	-	-
H3N3F1L1	-	-	-	-	-
H3N3F1E1	-	-	-	-	-
H5N3F1	-	-	-	-	-
H6N3	-	-	-	-	-
H5N3L1	-	-	-	-	-
H6N4	-	-	-	-	-
H5N4L1	-	-	-	-	-
H6N3E1	-	-	-	-	-
H4N5E1	-	-	-	-	-
H6N4L1	-	-	-	-	-
H4N7	-	-	-	-	-
H4N5F1E1	-	-	-	-	-
H5N5E1	-	-	-	-	-
H4N7E1	-	-	-	-	-
H5N5F1E1L1	-	-	-	-	-
H8N6F2	-	-	-	-	-
H10N6	-	-	-	-	-
H6N8L1	-	-	-	-	-
H6N5E2L1	-	-	-	-	-
H5N8E1L1	-	-	-	-	-
H6N5F2E1L2	-	-	-	-	-
H5N8F1E1L1	-	-	-	-	-
H4N7F2L3	-	-	-	-	-
H6N5F2E2L1	-	-	-	-	-
H7N6E1L2	-	-	-	-	-
H7N6E2L1	-	-	-	-	-
H7N6E3	-	-	-	-	-
H7N6F1E1L2	-	-	-	-	-
H7N6F1E2L1	-	-	-	-	-
*H7N6E1L3**	-	-	-	-	-
H7N6E2L2	-	-	-	-	-
H7N6E3L1	-	-	-	-	-
H7N6F1E1L3	-	-	-	-	-
H7N6F1E2L2	-	-	-	-	-
H7N6F1E3L1	-	-	-	-	-
H7N6F2E1L3	-	-	-	-	-
H7N6F2E2L2	-	-	-	-	-
H8N7E1L3	-	-	-	-	-
H8N7E2L2	-	-	-	-	-
H7N6F3E1L3	-	-	-	-	-
H8N7F1E1L3	-	-	-	-	-
H8N7F1E2L2	-	-	-	-	-
H9N8F1E1L2	-	-	-	-	-
H7N6F2E3L2	-	-	-	-	-
H9N8E2L2	-	-	-	-	-
H9N8E1L2	-	-	-	-	-
H8N7F2E2L2	-	-	-	-	-
H10N9E1L2	-	-	-	-	-
H10N9L4	-	-	-	-	-
H10N9E1L3	-	-	-	-	-
H10N9F1L4	-	-	-	-	-

Supplemental Table S4: TPNG Derived trait description. This table describes the calculation of each derived TPNG trait, lists a simple description and a graphical representation of the minimal glycan structure for each derived trait. The curly bracket in the graphical representation is used to indicate the part of the glycan that is represented by the derived trait, *e.g.* the galactose on the A2G trait indicates that it is the percentage of galactosylation that is represented.

Trait name	Description	Minimal glycan structure	Calculation
M	% High mannose compositions	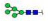	(1 * (H5N2 + H6N2 + H7N2 + H8N2 + H9N2)) / (1 * (H5N2 + H3N4 + H6N2 + H3N4F1 + H4N4 + H7N2 + H4N4F1 + H5N4 + H3N5F1 + H4N5 + H8N2 + H4N3F1E1 + H5N3E1 + H5N4F1 + H4N4E1 + H4N5F1 + H9N2 + H4N4F1E1 + H5N4E1 + H5N5F1 + H5N4L1F1 + H5N4F1E1 + H5N4L2 + H5N4E1L1 + H5N4E2 + H5N5F1E1 + H6N5E1 + H5N4F1L2 + H4N6F1E1 + H5N4F1E1L1 + H5N4F1E2 + H6N5F1E1 + H5N5E2 + H6N5L2 + H6N5E1L1 + H5N5F1E2 + H6N5E2 + H6N5F1E1L1 + H6N5E1L2 + H6N5E2L1 + H6N5E3 + H6N5F1L3 + H6N5F1E1L2 + H6N5F1E2L1 + H6N5F1E3))
Hy	% Hybrid compositions	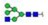	(1 * (H5N3E1)) / (1 * (H5N2 + H3N4 + H6N2 + H3N4F1 + H4N4 + H7N2 + H4N4F1 + H5N4 + H3N5F1 + H4N5 + H8N2 + H4N3F1E1 + H5N3E1 + H5N4F1 + H4N4E1 + H4N5F1 + H9N2 + H4N4F1E1 + H5N4E1 + H5N5F1 + H5N4L1F1 + H5N4F1E1 + H5N4L2 + H5N4E1L1 + H5N4E2 + H5N5F1E1 + H6N5E1 + H5N4F1L2 + H4N6F1E1 + H5N4F1E1L1 + H5N4F1E2 + H6N5F1E1 + H5N5E2 + H6N5L2 + H6N5E1L1 + H5N5F1E2 + H6N5E2 + H6N5F1E1L1 + H6N5E1L2 + H6N5E2L1 + H6N5E3 + H6N5F1L3 + H6N5F1E1L2 + H6N5F1E2L1 + H6N5F1E3))
C	% Complex compositions	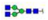	(1 * (H3N4 + H3N4F1 + H4N4 + H4N4F1 + H5N4 + H3N5F1 + H4N5 + H5N4F1 + H4N4E1 + H4N5F1 + H4N4F1E1 + H5N4E1 + H5N5F1 + H5N4L1F1 + H5N4F1E1 + H5N4L2 + H5N4E1L1 + H5N4E2 + H5N5F1E1 + H6N5E1 + H5N4F1L2 + H4N6F1E1 + H5N4F1E1L1 + H5N4F1E2 + H6N5F1E1 + H5N5E2 + H6N5L2 + H6N5E1L1 + H5N5F1E2 + H6N5E2 + H6N5F1E1L1 + H6N5E1L2 + H6N5E2L1 + H6N5E3 + H6N5F1L3 + H6N5F1E1L2 + H6N5F1E2L1 + H6N5F1E3)) / (1 * (H5N2 + H3N4 + H6N2 + H3N4F1 + H4N4 + H7N2 + H4N4F1 + H5N4 + H3N5F1 + H4N5 + H8N2 + H4N3F1E1 + H5N3E1 + H5N4F1 + H4N4E1 + H4N5F1 + H9N2 + H4N4F1E1 + H5N4E1 + H5N5F1 + H5N4L1F1 + H5N4F1E1 + H5N4L2 + H5N4E1L1 + H5N4E2 + H5N5F1E1 + H6N5E1 + H5N4F1L2 + H4N6F1E1 + H5N4F1E1L1 + H5N4F1E2 + H6N5F1E1 + H5N5E2 + H6N5L2 + H6N5E1L1 + H5N5F1E2 + H6N5E2 + H6N5F1E1L1 + H6N5E1L2 + H6N5E2L1 + H6N5E3 + H6N5F1L3 + H6N5F1E1L2 + H6N5F1E2L1 + H6N5F1E3))
A1	% Mono-antennary compositions		(1 * (H4N3F1E1)) / (1 * (H5N2 + H3N4 + H6N2 + H3N4F1 + H4N4 + H7N2 + H4N4F1 + H5N4 + H3N5F1 + H4N5 + H8N2 + H4N3F1E1 + H5N3E1 + H5N4F1 + H4N4E1 + H4N5F1 + H9N2 + H4N4F1E1 + H5N4E1 + H5N5F1 + H5N4L1F1 + H5N4F1E1 + H5N4L2 + H5N4E1L1 + H5N4E2 + H5N5F1E1 + H6N5E1 + H5N4F1L2 + H4N6F1E1 + H5N4F1E1L1 + H5N4F1E2 + H6N5F1E1 + H5N5E2 + H6N5L2 + H6N5E1L1 + H5N5F1E2 + H6N5E2 + H6N5F1E1L1 + H6N5E1L2 + H6N5E2L1 + H6N5E3 + H6N5F1L3 + H6N5F1E1L2 + H6N5F1E2L1 + H6N5F1E3))
A2	% Diantennary compositions	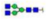	(1 * (H3N4 + H3N4F1 + H4N4 + H4N4F1 + H5N4 + H3N5F1 + H4N5 + H5N4F1 + H4N4E1 + H4N5F1 + H4N4F1E1 + H5N4E1 + H5N5F1 + H5N4L1F1 + H5N4F1E1 + H5N4L2 + H5N4E1L1 + H5N4E2 + H5N5F1E1 + H5N4F1L2 + H5N4F1E1L1 + H5N4F1E2 + H5N5E2 + H5N5F1E2)) / (1 * (H5N2 + H3N4 + H6N2 + H3N4F1 + H4N4 + H7N2 + H4N4F1 + H5N4 + H3N5F1 + H4N5 + H8N2 + H4N3F1E1 + H5N3E1 + H5N4F1 + H4N4E1 + H4N5F1 + H9N2 + H4N4F1E1 + H5N4E1 + H5N5F1 + H5N4L1F1 + H5N4F1E1 + H5N4L2 + H5N4E1L1 + H5N4E2 + H5N5F1E1 + H6N5E1 + H5N4F1L2 + H4N6F1E1 + H5N4F1E1L1 + H5N4F1E2 + H6N5F1E1 + H5N5E2 + H6N5L2 + H6N5E1L1 + H5N5F1E2 + H6N5E2 + H6N5F1E1L1 + H6N5E1L2 + H6N5E2L1 + H6N5E3 + H6N5F1L3 + H6N5F1E1L2 + H6N5F1E2L1 + H6N5F1E3))
A3	% Triantennary compositions	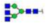	(1 * (H6N5E1 + H6N5F1E1 + H6N5L2 + H6N5E1L1 + H6N5E2 + H6N5F1E1L1 + H6N5E1L2 + H6N5E2L1 + H6N5E3 + H6N5F1L3 + H6N5F1E1L2 + H6N5F1E2L1 + H6N5F1E3)) / (1 * (H5N2 + H3N4 + H6N2 + H3N4F1 + H4N4 + H7N2 + H4N4F1 + H5N4 + H3N5F1 + H4N5 + H8N2 + H4N3F1E1 + H5N3E1 + H5N4F1 + H4N4E1 + H4N5F1 + H9N2 + H4N4F1E1 + H5N4E1 + H5N5F1 + H5N4L1F1 + H5N4F1E1 + H5N4L2 + H5N4E1L1 + H5N4E2 + H5N5F1E1 + H6N5E1 + H5N4F1L2 + H4N6F1E1 + H5N4F1E1L1 + H5N4F1E2 + H6N5F1E1 + H5N5E2 + H6N5L2 + H6N5E1L1 + H5N5F1E2 + H6N5E2 + H6N5F1E1L1 + H6N5E1L2 + H6N5E2L1 + H6N5E3 + H6N5F1L3 + H6N5F1E1L2 + H6N5F1E2L1 + H6N5F1E3))
B	% Bisection	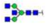	(1 * (H3N5F1 + H4N5 + H4N5F1 + H5N5F1 + H5N5F1E1 + H5N5E2 + H5N5F1E2)) / (1 * (H5N2 + H3N4 + H6N2 + H3N4F1 + H4N4 + H7N2 + H4N4F1 + H5N4 + H3N5F1 + H4N5 + H8N2 + H4N3F1E1 + H5N3E1 + H5N4F1 + H4N4E1 + H4N5F1 + H9N2 + H4N4F1E1 + H5N4E1 + H5N5F1 + H5N4L1F1 + H5N4F1E1 + H5N4L2 + H5N4E1L1 + H5N4E2 + H5N5F1E1 + H6N5E1 + H5N4F1L2 + H4N6F1E1 + H5N4F1E1L1 + H5N4F1E2 + H6N5F1E1 + H5N5E2 + H6N5L2 + H6N5E1L1 + H5N5F1E2 + H6N5E2 + H6N5F1E1L1 + H6N5E1L2 + H6N5E2L1 + H6N5E3 + H6N5F1L3 + H6N5F1E1L2 + H6N5F1E2L1 + H6N5F1E3))
F	% Fucosylation		(1 * (H3N4F1 + H4N4F1 + H3N5F1 + H4N3F1E1 + H5N4F1 + H4N5F1 + H4N4F1E1 + H5N5F1 + H5N4L1F1 + H5N4F1E1 + H5N5F1E1 + H5N4F1L2 + H4N6F1E1 + H5N4F1E1L1 + H5N4F1E2 + H6N5F1E1 + H5N5F1E2 + H6N5F1E1L1 + H6N5F1L3 + H6N5F1E1L2 + H6N5F1E2L1 + H6N5F1E3)) / (1 * (H5N2 + H3N4 + H6N2 + H3N4F1 + H4N4 + H7N2 + H4N4F1 + H5N4 + H3N5F1 + H4N5 + H8N2 + H4N3F1E1 + H5N3E1 + H5N4F1 + H4N4E1 + H4N5F1 + H9N2 + H4N4F1E1 + H5N4E1 + H5N5F1 + H5N4L1F1 + H5N4F1E1 + H5N4L2 + H5N4E1L1 + H5N4E2 + H5N5F1E1 + H6N5E1 + H5N4F1L2 + H4N6F1E1 + H5N4F1E1L1 + H5N4F1E2 + H6N5F1E1 + H5N5E2 + H6N5L2 + H6N5E1L1 + H5N5F1E2 + H6N5E2 + H6N5F1E1L1 + H6N5E1L2 + H6N5E2L1 + H6N5E3 + H6N5F1L3 + H6N5F1E1L2 + H6N5F1E2L1 + H6N5F1E3))
MM	Average number of mannoses on high mannose compositions	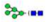	(5 * (H5N2) + 6 * (H6N2) + 7 * (H7N2) + 8 * (H8N2) + 9 * (H9N2)) / (1 * (H5N2 + H6N2 + H7N2 + H8N2 + H9N2))
A2F	% Fucosylation of diantennary compositions	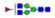	(1 * (H3N4F1 + H4N4F1 + H3N5F1 + H5N4F1 + H4N5F1 + H4N4F1E1 + H5N5F1 + H5N4L1F1 + H5N4F1E1 + H5N5F1E1 + H5N4F1L2 + H5N4F1E1L1 + H5N4F1E2 + H5N5F1E2)) / (1 * (H3N4 + H3N4F1 + H4N4 + H4N4F1 + H5N4 + H3N5F1 + H4N5 + H5N4F1 + H4N4E1 + H4N5F1 + H4N4F1E1 + H5N4E1 + H5N5F1 + H5N4L1F1 + H5N4F1E1 + H5N4L2 + H5N4E1L1 + H5N4E2 + H5N5F1E1 + H5N4F1L2 + H5N4F1E1L1 + H5N4F1E2 + H5N5E2 + H5N5F1E2))
A3F	% Fucosylation of triantennary compositions	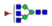	(1 * (H6N5F1E1 + H6N5F1E1L1 + H6N5F1L3 + H6N5F1E1L2 + H6N5F1E2L1 + H6N5F1E3)) / (1 * (H6N5E1 + H6N5F1E1 + H6N5L2 + H6N5E1L1 + H6N5E2 + H6N5F1E1L1 + H6N5E1L2 + H6N5E2L1 + H6N5E3 + H6N5F1L3 + H6N5F1E1L2 + H6N5F1E2L1 + H6N5F1E3))
A2B	% Bisection of diantennary compositions	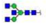	(1 * (H3N5F1 + H4N5 + H4N5F1 + H5N5F1 + H5N5F1E1 + H5N5E2 + H5N5F1E2)) / (1 * (H3N4 + H3N4F1 + H4N4 + H4N4F1 + H5N4 + H3N5F1 + H4N5 + H5N4F1 + H4N4E1 + H4N5F1 + H4N4F1E1 + H5N4E1 + H5N5F1 + H5N4L1F1 + H5N4F1E1 + H5N4L2 + H5N4E1L1 + H5N4E2 + H5N5F1E1 + H5N4F1L2 + H5N4F1E1L1 + H5N4F1E2 + H5N5E2 + H5N5F1E2))
A2F0B	% Bisection of non-fucosylated diantennary compositions	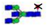	(1 * (H4N5 + H5N5E2)) / (1 * (H3N4 + H4N4 + H5N4 + H4N5 + H4N4E1 + H5N4E1 + H5N4L2 + H5N4E1L1 + H5N4E2 + H5N5E2))
A2FB	% Bisection of fucosylated diantennary compositions	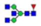	(1 * (H3N5F1 + H4N5F1 + H5N5F1 + H5N5F1E1 + H5N5F1E2)) / (1 * (H3N4F1 + H4N4F1 + H3N5F1 + H5N4F1 + H4N5F1 + H4N4F1E1 + H5N5F1 + H5N4L1F1 + H5N4F1E1 + H5N5F1E1 + H5N4F1L2 + H5N4F1E1L1 + H5N4F1E2 + H5N5F1E2))

Trait name	Description	Minimal glycan structure	Calculation
A2G	% Galactosylation of diantennary compositions	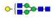	(1/2 * (H4N4 + H4N4F1 + H4N5 + H4N4E1 + H4N5F1 + H4N4F1E1) + 2/2 * (H5N4 + H5N4F1 + H5N4E1 + H5N5F1 + H5N4L1F1 + H5N4F1E1 + H5N4L2 + H5N4E1L1 + H5N4E2 + H5N5F1E1 + H5N4F1L2 + H5N4F1E1L1 + H5N4F1E2 + H5N5E2 + H5N5F1E2)) / (1 * (H3N4 + H3N4F1 + H4N4 + H4N4F1 + H5N4 + H3N5F1 + H4N5 + H5N4F1 + H4N4E1 + H4N5F1 + H4N4F1E1 + H5N4E1 + H5N5F1 + H5N4L1F1 + H5N4F1E1 + H5N4L2 + H5N4E1L1 + H5N4E2 + H5N5F1E1 + H5N4F1L2 + H5N4F1E1L1 + H5N4F1E2 + H5N5E2 + H5N5F1E2))
A3G	% Galactosylation of triantennary compositions	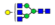	(1/3 * (0) + 2/3 * (0) + 3/3 * (H6N5E1 + H6N5F1E1 + H6N5L2 + H6N5E1L1 + H6N5E2 + H6N5F1E1L1 + H6N5E1L2 + H6N5E2L1 + H6N5E3 + H6N5F1L3 + H6N5F1E1L2 + H6N5F1E2L1 + H6N5F1E3)) / (1 * (H6N5E1 + H6N5F1E1 + H6N5L2 + H6N5E1L1 + H6N5E2 + H6N5F1E1L1 + H6N5E1L2 + H6N5E2L1 + H6N5E3 + H6N5F1L3 + H6N5F1E1L2 + H6N5F1E2L1 + H6N5F1E3))
A2F0G	% Galactosylation of non-fucosylated diantennary compositions	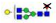	(1/2 * (H4N4 + H4N5 + H4N4E1) + 2/2 * (H5N4 + H5N4E1 + H5N4L2 + H5N4E1L1 + H5N4E2 + H5N5E2)) / (1 * (H3N4 + H4N4 + H5N4 + H4N5 + H4N4E1 + H5N4E1 + H5N4L2 + H5N4E1L1 + H5N4E2 + H5N5E2))
A3F0G	% Galactosylation of non-fucosylated triantennary compositions	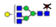	(1/3 * (0) + 2/3 * (0) + 3/3 * (H6N5E1 + H6N5L2 + H6N5E1L1 + H6N5E2 + H6N5E1L2 + H6N5E2L1 + H6N5E3)) / (1 * (H6N5E1 + H6N5L2 + H6N5E1L1 + H6N5E2 + H6N5E1L2 + H6N5E2L1 + H6N5E3))
A2FG	% Galactosylation of fucosylated diantennary compositions	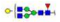	(1/2 * (H4N4F1 + H4N5F1 + H4N4F1E1) + 2/2 * (H5N4F1 + H5N5F1 + H5N4L1F1 + H5N4F1E1 + H5N5F1E1 + H5N4F1L2 + H5N4F1E1L1 + H5N4F1E2 + H5N5F1E2)) / (1 * (H3N4F1 + H4N4F1 + H3N5F1 + H5N4F1 + H4N5F1 + H4N4F1E1 + H5N5F1 + H5N4L1F1 + H5N4F1E1 + H5N5F1E1 + H5N4F1L2 + H5N4F1E1L1 + H5N4F1E2 + H5N5F1E2))
A3FG	% Galactosylation of fucosylated triantennary compositions	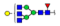	(1/3 * (0) + 2/3 * (0) + 3/3 * (H6N5F1E1 + H6N5F1E1L1 + H6N5F1L3 + H6N5F1E1L2 + H6N5F1E2L1 + H6N5F1E3)) / (1 * (H6N5F1E1 + H6N5F1E1L1 + H6N5F1L3 + H6N5F1E1L2 + H6N5F1E2L1 + H6N5F1E3))
A2S	% Sialylation of diantennary compositions	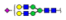	(1/2 * (H4N4E1 + H4N4F1E1 + H5N4E1 + H5N4L1F1 + H5N4F1E1 + H5N5F1E1) + 2/2 * (H5N4L2 + H5N4E1L1 + H5N4E2 + H5N4F1L2 + H5N4F1E1L1 + H5N4F1E2 + H5N5E2 + H5N5F1E2)) / (1 * (H3N4 + H3N4F1 + H4N4 + H4N4F1 + H5N4 + H3N5F1 + H4N5 + H5N4F1 + H4N4E1 + H4N5F1 + H4N4F1E1 + H5N4E1 + H5N5F1 + H5N4L1F1 + H5N4F1E1 + H5N4L2 + H5N4E1L1 + H5N4E2 + H5N5F1E1 + H5N4F1L2 + H5N4F1E1L1 + H5N4F1E2 + H5N5E2 + H5N5F1E2)).
A3S	% Sialylation of triantennary compositions	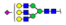	(1/3 * (H6N5E1 + H6N5F1E1) + 2/3 * (H6N5L2 + H6N5E1L1 + H6N5E2 + H6N5F1E1L1) + 3/3 * (H6N5E1L2 + H6N5E2L1 + H6N5E3 + H6N5F1L3 + H6N5F1E1L2 + H6N5F1E2L1 + H6N5F1E3)) / (1 * (H6N5E1 + H6N5F1E1 + H6N5L2 + H6N5E1L1 + H6N5E2 + H6N5F1E1L1 + H6N5E1L2 + H6N5E2L1 + H6N5E3 + H6N5F1L3 + H6N5F1E1L2 + H6N5F1E2L1 + H6N5F1E3))
A2F0S	% Sialylation of non-fucosylated diantennary compositions	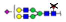	(1/2 * (H4N4E1 + H5N4E1) + 2/2 * (H5N4L2 + H5N4E1L1 + H5N4E2 + H5N5E2)) / (1 * (H3N4 + H4N4 + H5N4 + H4N5 + H4N4E1 + H5N4E1 + H5N4L2 + H5N4E1L1 + H5N4E2 + H5N5E2))
A3F0S	% Sialylation of non-fucosylated triantennary compositions	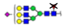	(1/3 * (H6N5E1) + 2/3 * (H6N5L2 + H6N5E1L1 + H6N5E2) + 3/3 * (H6N5E1L2 + H6N5E2L1 + H6N5E3)) / (1 * (H6N5E1 + H6N5L2 + H6N5E1L1 + H6N5E2 + H6N5E1L2 + H6N5E2L1 + H6N5E3))
A2FS	% Sialylation of fucosylated diantennary compositions	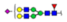	(1/2 * (H4N4F1E1 + H5N4L1F1 + H5N4F1E1 + H5N5F1E1) + 2/2 * (H5N4F1L2 + H5N4F1E1L1 + H5N4F1E2 + H5N5F1E2)) / (1 * (H3N4F1 + H4N4F1 + H3N5F1 + H5N4F1 + H4N5F1 + H4N4F1E1 + H5N5F1 + H5N4L1F1 + H5N4F1E1 + H5N5F1E1 + H5N4F1L2 + H5N4F1E1L1 + H5N4F1E2 + H5N5F1E2))
A3FS	% Sialylation of fucosylated triantennary compositions	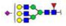	(1/3 * (H6N5F1E1) + 2/3 * (H6N5F1E1L1) + 3/3 * (H6N5F1L3 + H6N5F1E1L2 + H6N5F1E2L1 + H6N5F1E3)) / (1 * (H6N5F1E1 + H6N5F1E1L1 + H6N5F1L3 + H6N5F1E1L2 + H6N5F1E2L1 + H6N5F1E3))
A2L	% α2,3-linked sialylation of diantennary compositions	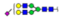	(1/2 * (H5N4L1F1 + H5N4E1L1 + H5N4F1E1L1) + 2/2 * (H5N4L2 + H5N4F1L2)) / (1 * (H3N4 + H3N4F1 + H4N4 + H4N4F1 + H5N4 + H3N5F1 + H4N5 + H5N4F1 + H4N4E1 + H4N5F1 + H4N4F1E1 + H5N4E1 + H5N5F1 + H5N4L1F1 + H5N4F1E1 + H5N4L2 + H5N4E1L1 + H5N4E2 + H5N5F1E1 + H5N4F1L2 + H5N4F1E1L1 + H5N4F1E2 + H5N5E2 + H5N5F1E2))
A3L	% α2,3-linked sialylation of triantennarycompositions	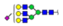	(1/3 * (H6N5E1L1 + H6N5F1E1L1 + H6N5E2L1 + H6N5F1E2L1) + 2/3 * (H6N5L2 + H6N5E1L2 + H6N5F1E1L2) + 3/3 * (H6N5F1L3)) / (1 * (H6N5E1 + H6N5F1E1 + H6N5L2 + H6N5E1L1 + H6N5E2 + H6N5F1E1L1 + H6N5E1L2 + H6N5E2L1 + H6N5E3 + H6N5F1L3 + H6N5F1E1L2 + H6N5F1E2L1 + H6N5F1E3))
A2F0L	% α2,3-linked sialylation of non-fucosylated diantennary compositions	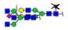	(1/2 * (H5N4E1L1) + 2/2 * (H5N4L2)) / (1 * (H3N4 + H4N4 + H5N4 + H4N5 + H4N4E1 + H5N4E1 + H5N4L2 + H5N4E1L1 + H5N4E2 + H5N5E2))
A3F0L	% α2,3-linked sialylation of non-fucosylated triantennary compositions	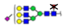	(1/3 * (H6N5E1L1 + H6N5E2L1) + 2/3 * (H6N5L2 + H6N5E1L2) + 3/3 * (0)) / (1 * (H6N5E1 + H6N5L2 + H6N5E1L1 + H6N5E2 + H6N5E1L2 + H6N5E2L1 + H6N5E3))
A2FL	% α2,3-linked sialylation of fucosylated diantennary compositions	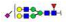	(1/2 * (H5N4L1F1 + H5N4F1E1L1) + 2/2 * (H5N4F1L2)) / (1 * (H3N4F1 + H4N4F1 + H3N5F1 + H5N4F1 + H4N5F1 + H4N4F1E1 + H5N5F1 + H5N4L1F1 + H5N4F1E1 + H5N5F1E1 + H5N4F1L2 + H5N4F1E1L1 + H5N4F1E2 + H5N5F1E2))
A3FL	% α2,3-linked sialylation of fucosylated triantennary compositions	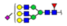	(1/3 * (H6N5F1E1L1 + H6N5F1E2L1) + 2/3 * (H6N5F1E1L2) + 3/3 * (H6N5F1L3)) / (1 * (H6N5F1E1 + H6N5F1E1L1 + H6N5F1L3 + H6N5F1E1L2 + H6N5F1E2L1 + H6N5F1E3))
A2E	% α2,6-linked sialylation of diantennary compositions	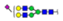	(1/2 * (H4N4E1 + H4N4F1E1 + H5N4E1 + H5N4F1E1 + H5N4E1L1 + H5N5F1E1 + H5N4F1E1L1) + 2/2 * (H5N4E2 + H5N4F1E2 + H5N5E2 + H5N5F1E2)) / (1 * (H3N4 + H3N4F1 + H4N4 + H4N4F1 + H5N4 + H3N5F1 + H4N5 + H5N4F1 + H4N4E1 + H4N5F1 + H4N4F1E1 + H5N4E1 + H5N5F1 + H5N4L1F1 + H5N4F1E1 + H5N4L2 + H5N4E1L1 + H5N4E2 + H5N5F1E1 + H5N4F1L2 + H5N4F1E1L1 + H5N4F1E2 + H5N5E2 + H5N5F1E2))
A3E	% α2,6-linked sialylation of triantennary compositions	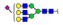	(1/3 * (H6N5E1 + H6N5F1E1 + H6N5E1L1 + H6N5F1E1L1 + H6N5E1L2 + H6N5F1E1L2) + 2/3 * (H6N5E2 + H6N5E2L1 + H6N5F1E2L1) + 3/3 * (H6N5E3 + H6N5F1E3)) / (1 * (H6N5E1 + H6N5F1E1 + H6N5L2 + H6N5E1L1 + H6N5E2 + H6N5F1E1L1 + H6N5E1L2 + H6N5E2L1 + H6N5E3 + H6N5F1L3 + H6N5F1E1L2 + H6N5F1E2L1 + H6N5F1E3))

Trait name	Description	Minimal glycan structure	Calculation
A2F0E	% α2,6-linked sialylation of non-fucosylated diantennary compositions	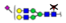	(1/2 * (H4N4E1 + H5N4E1 + H5N4E1L1) + 2/2 * (H5N4E2 + H5N5E2)) / (1 * (H3N4 + H4N4 + H5N4 + H4N5 + H4N4E1 + H5N4E1 + H5N4L2 + H5N4E1L1 + H5N4E2 + H5N5E2))
A3F0E	% α2,6-linked sialylatlion of non-fucosylated triantennary compositions	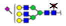	(1/3 * (H6N5E1 + H6N5E1L1 + H6N5E1L2) + 2/3 * (H6N5E2 + H6N5E2L1) + 3/3 * (H6N5E3)) / (1 * (H6N5E1 + H6N5L2 + H6N5E1L1 + H6N5E2 + H6N5E1L2 + H6N5E2L1 + H6N5E3))
A2FE	% α2,6-linked sialylation of fucosylated diantennary compositions	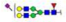	(1/2 * (H4N4F1E1 + H5N4F1E1 + H5N5F1E1 + H5N4F1E1L1) + 2/2 * (H5N4F1E2 + H5N5F1E2)) / (1 * (H3N4F1 + H4N4F1 + H3N5F1 + H5N4F1 + H4N5F1 + H4N4F1E1 + H5N5F1 + H5N4L1F1 + H5N4F1E1 + H5N5F1E1 + H5N4F1L2 + H5N4F1E1L1 + H5N4F1E2 + H5N5F1E2))
A3FE	% α2,6-linked sialylation of fucosylated triantennary compositions	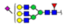	(1/3 * (H6N5F1E1 + H6N5F1E1L1 + H6N5F1E1L2) + 2/3 * (H6N5F1E2L1) + 3/3 * (H6N5F1E3)) / (1 * (H6N5F1E1 + H6N5F1E1L1 + H6N5F1L3 + H6N5F1E1L2 + H6N5F1E2L1 + H6N5F1E3))
A2GS	% Sialylation per galactose of diantennary compositions	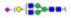	(1 * (A2S)) / (1 * (A2G))
A3GS	% Sialylation per galactose of triantennary compositions	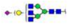	(1 * (A3S)) / (1 * (A3G))
A2F0GS	% Sialylation per galactose of non-fucosylated diantennary compositions	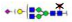	(1 * (A2F0S)) / (1 * (A2F0G))
A3F0GS	% Sialylation per galactose of non-fucosylated triantennary compositions	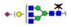	(1 * (A3F0S)) / (1 * (A3F0G))
A2FGS	% Sialylation per galactose of fucosylated diantennary compositions	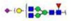	(1 * (A2FS)) / (1 * (A2FG))
A3FGS	% Sialylation per galactose of fucosylated triantennary compositions	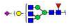	(1 * (A3FS)) / (1 * (A3FG))
A2GL	% α2,3-linked sialylation per galactose on diantennary compositions	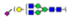	(1 * (A2L)) / (1 * (A2G))
A3GL	% α2,3-linked sialylation per galactose of triantennary compositions	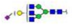	(1 * (A3L)) / (1 * (A3G))
A2F0GL	% α2,3-linked sialylation per galactose of non-fucosylated diantennary compositions	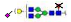	(1 * (A2F0L)) / (1 * (A2F0G))
A3F0GL	% α2,3-linked sialylation per galactose of non-fucosylated triantennary compositions	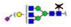	(1 * (A3F0L)) / (1 * (A3F0G))
A2FGL	% α2,3-linked sialylation per galactose of fucosylated diantennary compositions	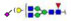	(1 * (A2FL)) / (1 * (A2FG))
A3FGL	% α2,3-linked sialylation per galactose of fucosylated triantennary compositions	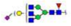	(1 * (A3FL)) / (1 * (A3FG))
A2GE	% α2,6-linked sialylation per galactose of diantennary compositions	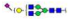	(1 * (A2E)) / (1 * (A2G))
A3GE	% α2,6-linked sialylation per galactose of triantennary compositions	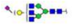	(1 * (A3E)) / (1 * (A3G))
A2F0GE	% α2,6-linked sialylation per galactose of non-fucosylated diantennary compositions	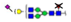	(1 * (A2F0E)) / (1 * (A2F0G))
A3F0GE	% α2,6-linked sialylation per galactose of non-fucosylated triantennary compositions	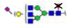	(1 * (A3F0E)) / (1 * (A3F0G))
A2FGE	% α2,6-linked sialylation per galactose of fucosylated diantennary compositions	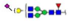	(1 * (A2FE)) / (1 * (A2FG))

Trait name	Description	Minimal glycan structure	Calculation
A3FGE	% α2,6-linked sialylation per galactose of fucosylated triantennary compositions	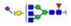	(1 * (A3FE)) / (1 * (A3FG))
AG	% Galactosylation per antenna		(A2 * (A2G) + A3 * (A3G)) / (1 * (A2 + A3))
AS	% Sialylation per antenna		(A2 * (A2S) + A3 * (A3S)) / (1 * (A2 + A3))
AL	% α2,3-linked sialylation per antenna		(A2 * (A2L) + A3 * (A3L)) / (1 * (A2 + A3))
AE	% α2,6-linked sialylation per antenna	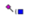	(A2 * (A2E) + A3 * (A3E)) / (1 * (A2 + A3))
GS	% Sialylation per galactose		(A2 * (A2GS) + A3 * (A3GS)) / (1 * (A2 + A3))
GL	% α2,3-linked sialylation per galactose		(A2 * (A2GL) + A3 * (A3GL)) / (1 * (A2 + A3))
GE	% α2,6-linked sialylation per galactose		(A2 * (A2GE) + A3 * (A3GE)) / (1 * (A2 + A3))
A2GRle	% Sialylation of diantennary compositions being α2,3-linked	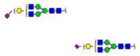	(1 * (A2GL)) / (1 * (A2GL + A2GE))
A2F0GRle	% Sialylation of non-fucosylated diantennary compositions being α2,3-linked	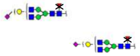	(1 * (A2F0GL)) / (1 * (A2F0GL + A2F0GE))
A2FGRle	% Sialylation of fucosylated diantennary compositions being α2,3-linked	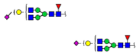	(1 * (A2FGL)) / (1 * (A2FGL + A2FGE))
A3GRle	% Sialylation of triantennary compositions being α2,3-linked	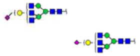	(1 * (A3GL)) / (1 * (A3GL + A3GE))
A3F0GRle	% Sialylation of non-fucosylated triantennary compositions being α2,3-linked	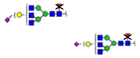	(1 * (A3F0GL)) / (1 * (A3F0GL + A3F0GE))
A3FGRle	% Sialylation of fucosylated triantennary compositions being α2,3-linked	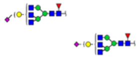	(1 * (A3FGL)) / (1 * (A3FGL + A3FGE))
AGRle	% Sialylation of complex compositions being α2,3-linked	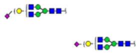	(1 * (GL)) / (1 * (GL + GE))
IGG	% Glycan compositions unique to IgG	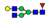	(H3N4F1 + H4N4F1 + H5N4F1 + H3N5F1 + H4N5F1 + H5N5F1)

Supplemental Table S5: IgG glycan accurate mass determination. The exact mass, average mass error and corresponding average accurate masses are presented for all glycans that were quantified in the IgG samples. The abbreviations used in this table are M for maternal, UC for umbilical cord, ME for mass error and Acc. for accurate.

Glycan	Exact Mass	M. ME [ppm]	UC ME [ppm]	M. Acc. Mass	UC Acc. Mass
H5N2	1257.42	-16.10	-7.07	1257.40	1257.41
H3N4	1339.48	-147.40	-14.44	1339.28	1339.46
H6N2	1419.48	-77.72	-18.02	1419.37	1419.45
H3N4F1	1485.53	0.38	-1.48	1485.53	1485.53
H4N4	1501.53	27.67	2.05	1501.57	1501.53
H3N5	1542.56	10.20	8.47	1542.57	1542.57
H7N2	1581.53	48.80	18.12	1581.61	1581.56
H4N4F1	1647.59	-0.55	-0.27	1647.59	1647.59
H5N4	1663.58	-3.37	1.20	1663.58	1663.58
H3N5F1	1688.61	-1.88	-2.08	1688.61	1688.61
H4N5	1704.61	-18.08	-2.78	1704.58	1704.60
H8N2	1743.58	-12.91	-19.51	1743.56	1743.55
H4N4L1	1774.61	-13.77	6.70	1774.59	1774.63
H5N4F1	1809.64	0.08	0.93	1809.64	1809.64
H4N4E1	1820.66	-17.16	-62.85	1820.62	1820.54
H4N5F1	1850.67	-0.42	3.08	1850.67	1850.67
H5N5	1866.66	-48.64	-14.10	1866.57	1866.63
H9N2	1905.63	-18.34	-45.68	1905.60	1905.55
H5N4L1	1936.67	-39.89	13.55	1936.59	1936.69
H4N4F1E1	1966.71	0.40	1.84	1966.71	1966.72
H5N4E1	1982.71	-1.14	1.48	1982.71	1982.71
H5N5F1	2012.72	2.93	0.12	2012.72	2012.72
H4N5E1	2023.73	24.18	19.14	2023.78	2023.77
H5N4F1L1	2082.72	15.04	27.48	2082.76	2082.78
H5N4F1E1	2128.77	-0.17	1.18	2128.77	2128.77
H4N5F1E1	2169.79	11.76	16.33	2169.82	2169.83
H5N5E1	2185.79	25.35	14.81	2185.84	2185.82
H5N4E1L1	2255.79	31.32	19.22	2255.86	2255.84
H5N5F1L1	2285.80	33.83	19.66	2285.88	2285.85
H5N4E2	2301.83	2.13	3.83	2301.84	2301.84
H5N5F1E1	2331.85	-2.92	-1.55	2331.84	2331.84
H5N4F1E1L1	2401.85	7.24	6.48	2401.87	2401.87
H5N4F1E2	2447.89	-2.74	-1.74	2447.89	2447.89
H5N5E2	2504.91	-1.25	-14.73	2504.91	2504.88
H5N5F1L2	2558.89	7.90	30.87	2558.91	2558.97
H5N5F1E1L1	2604.93	14.09	28.02	2604.97	2605.00
H5N5F1E2	2650.97	1.55	-8.13	2650.98	2650.95

Supplemental Table S6: TPNG glycan accurate mass determination. The exact mass, average mass error and corresponding average accurate masses are presented for all glycans that were quantified in the TPNG samples. The abbreviations used in this table are M for maternal, UC for umbilical cord, ME for mass error and Acc. for accurate.

Glycan	Exact Mass	M. ME [ppm]	UC ME [ppm]	M. Acc. Mass	UC Acc. Mass
H5N2	1257.42	-14.61	-16.37	1257.40	1257.40
H3N4	1339.48	51.61	16.88	1339.54	1339.50
H6N2	1419.48	-6.86	3.21	1419.47	1419.48
H3N4F1	1485.53	2.50	-2.87	1485.54	1485.53
H4N4	1501.53	45.85	15.46	1501.60	1501.55
H7N2	1581.53	-0.52	9.06	1581.53	1581.54
H4N4F1	1647.59	0.13	0.03	1647.59	1647.59
H5N4	1663.58	0.70	1.17	1663.58	1663.58
H3N5F1	1688.61	-15.21	8.25	1688.59	1688.63
H4N5	1704.61	-8.08	14.19	1704.59	1704.63
H8N2	1743.58	-3.93	-3.80	1743.57	1743.57
H4N3F1E1	1763.63	11.26	26.05	1763.65	1763.68
H5N3E1	1779.63	19.48	20.19	1779.66	1779.66
H5N4F1	1809.64	1.32	1.04	1809.64	1809.64
H4N4E1	1820.66	18.89	1.77	1820.69	1820.66
H4N5F1	1850.67	2.58	1.81	1850.67	1850.67
H9N2	1905.63	-3.13	-4.63	1905.63	1905.63
H4N4F1E1	1966.71	-3.68	3.89	1966.71	1966.72
H5N4E1	1982.71	0.85	1.03	1982.71	1982.71
H5N5F1	2012.72	4.02	-0.17	2012.73	2012.72
H5N4L1F1	2082.72	6.67	-1.73	2082.74	2082.72
H5N4F1E1	2128.77	-0.77	-1.08	2128.76	2128.76
H5N4L2	2209.75	-1.32	49.53	2209.75	2209.86
H5N4E1L1	2255.79	0.27	2.72	2255.79	2255.80
H5N4E2	2301.83	-1.32	-0.53	2301.83	2301.83
H5N5F1E1	2331.85	-2.40	-1.68	2331.84	2331.84
H6N5E1	2347.84	0.88	-12.04	2347.84	2347.81
H5N4F1L2	2355.81	3.62	12.93	2355.82	2355.84
H4N6F1E1	2372.87	-10.67	7.71	2372.85	2372.89
H5N4F1E1L1	2401.85	-0.40	-1.26	2401.85	2401.85
H5N4F1E2	2447.89	-2.46	-0.70	2447.89	2447.89
H6N5F1E1	2493.90	-9.09	16.57	2493.88	2493.94
H5N5E2	2504.91	-5.67	-2.87	2504.90	2504.91
H6N5L2	2574.88	8.29	-5.43	2574.90	2574.87
H6N5E1L1	2620.93	-4.87	2.84	2620.91	2620.93
H5N5F1E2	2650.97	-3.69	-4.33	2650.96	2650.96
H6N5E2	2666.97	2.49	-1.04	2666.97	2666.96
H6N5F1E1L1	2766.98	-4.52	7.59	2766.97	2767.00
H6N5E1L2	2894.01	4.89	14.93	2894.02	2894.05
H6N5E2L1	2940.05	-1.40	2.33	2940.05	2940.06
H6N5E3	2986.09	-4.25	-3.87	2986.08	2986.08
H6N5F1L3	2994.03	10.35	8.91	2994.06	2994.05
H6N5F1E1L2	3040.07	0.71	-2.79	3040.07	3040.06
H6N5F1E2L1	3086.11	-3.19	-0.76	3086.10	3086.11
H6N5F1E3	3132.15	2.64	8.46	3132.16	3132.18
